# Automatic segmentation of the striatum and globus pallidus using MIST: Multimodal Image Segmentation Tool

**DOI:** 10.1016/j.neuroimage.2015.10.013

**Published:** 2016-01-15

**Authors:** Eelke Visser, Max C. Keuken, Gwenaëlle Douaud, Veronique Gaura, Anne-Catherine Bachoud-Levi, Philippe Remy, Birte U. Forstmann, Mark Jenkinson

**Affiliations:** a FMRIB Centre, Nuffield Department of Clinical Neurosciences, University of Oxford, Oxford, United Kingdom; b Amsterdam Brain and Cognition, University of Amsterdam, Amsterdam, Netherlands; c Commissariat à l'Energie Atomique et aux Energies Alternatives (CEA), Département des Sciences du Vivant (DSV), Institut d'Imagerie Biomédicale (I2BM), MIRCen, F-92260 Fontenay-aux-Roses, France; d Centre National de la Recherche Scientifique (CNRS), Université Paris-Sud, Université Paris-Saclay, UMR 9199, Neurodegenerative Diseases Laboratory, F-92260 Fontenay-aux-Roses, France; e AP-HP, Hôpital Henri Mondor, Centre de Référence-Maladie de Huntington, Neurologie cognitive, Créteil, France; f Université Paris Est, Faculté de médecine, Créteil, France; g INSERM U955, Equipe 01, Neuropsychologie Interventionnelle, Créteil, France; h Département d'Etudes Cognitives, Ecole Normale Supérieure, PSL Research University, Paris, France; i Centre Expert Parkinson et NEURATRIS, CHU Henri Mondor, Pôle Neuro-Locomoteur, Assistance Publique Hôpitaux de Paris et Université Paris Est Créteil, France

**Keywords:** Segmentation, Multimodal, Striatum, Globus pallidus, Brain, Huntington

## Abstract

Accurate segmentation of the subcortical structures is frequently required in neuroimaging studies. Most existing methods use only a *T*_1_-weighted MRI volume to segment all supported structures and usually rely on a database of training data. We propose a new method that can use multiple image modalities simultaneously and a single reference segmentation for initialisation, without the need for a manually labelled training set. The method models intensity profiles in multiple images around the boundaries of the structure after nonlinear registration. It is trained using a set of unlabelled training data, which may be the same images that are to be segmented, and it can automatically infer the location of the physical boundary using user-specified priors. We show that the method produces high-quality segmentations of the striatum, which is clearly visible on *T*_1_-weighted scans, and the globus pallidus, which has poor contrast on such scans. The method compares favourably to existing methods, showing greater overlap with manual segmentations and better consistency.

## Highlights

• We describe a new multimodal method for subcortical segmentation and apply it to the striatum and globus pallidus.
• The method does not require multiple manual segmentations for training.
• The method improves upon exiting methods that only use a *T*_1_‐weighted image (FIRST and FreeSurfer).
• Multimodal segmentation is particularly advantageous for the globus pallidus, which has poor contrast on *T*_1_‐weighted scans.

## Introduction

The subcortical structures of the brain are a set of nuclei with unique anatomical shapes and, due to their histological composition, specific MR contrast properties. Automatic segmentation of these structures is often desired, as manually segmenting them is a process that is both laborious and prone to labelling inconsistencies and errors. Unlike cortical segmentation, which is a nearly universal process for all of the cortex, subcortical segmentation requires methods that are tailored to these structures specifically, due to both their unique anatomies and contrasts. These methods produce an estimate of the extent of the structures, either as voxel labels or in the form of a mesh describing the outer surface of the shape.

Numerous methods have been proposed to automatically segment the subcortical structures and a number of these are used routinely in imaging studies. The input data typically consist of a single *T*_1_-weighted magnetic resonance imaging (MRI) volume. Such an image is often a standard part of the acquisition protocol in neuroimaging studies and serves as the anatomical reference in the analysis. The *T*_1_-weighted contrast is most suitable for structures such as the putamen and the caudate nucleus, which are clearly visible on such scans. Other structures, like the globus pallidus and the red nucleus are barely visible or invisible. In such cases, a segmentation method that only uses a *T*_1_-weighted volume needs to rely more on anatomical priors.

Iosifescu et al. (1997), Khan et al. (2005) and Lin et al. (2010) describe methods that segment the subcortical structures by non-linearly registering an atlas of the subcortical anatomy to the new scan. This idea is extended by Gouttard et al. (2007) through the use of a probabilistic atlas. Yan et al. (2013) describe an adaptive atlas and Van Leemput (2009) applies Bayesian inference to optimise atlas and registration parameters during atlas generation. Multiple manual segmentations can be individually registered to a new scan and combined into a final segmentation by building a probabilistic atlas (Svarer et al., 2005) or using a decision fusion scheme such as using the modal label (Heckemann et al., 2006), support vector machine-based selection (Hao et al., 2014), graph-based selection (Wang et al., 2014), joint label fusion (Wang et al., 2013, Yushkevich et al., 2015) or fusion of the likelihoods under the different atlases (Tang et al., 2013). J. Morra et al. (2008a) and J.H. Morra et al. (2008b) use a classifier trained on non-local image features using an extension to AdaBoost (Freund and Schapire, 1997). A method using a single template and explicit modelling of an hierarchy of structures is described by Wu and Chung (2009).

Fischl et al., 2002, Fischl et al., 2004 and Han and Fischl (2007) take a different approach in the method that is used in the FreeSurfer software package (http://surfer.nmr.mgh.harvard.edu/). A probabilistic atlas, which is derived from a number of manual segmentations, is still used, but in this case it is used as a prior on a Markov random field (MRF). The spatial constraints imposed by the atlas allow the MRF to segment the image into the large number of classes needed to segment the subcortical structures. This idea is combined with a multi-atlas approach by van der Lijn et al. (2008) and Wolz and Aljabar (2009). A mesh-based spatial prior can also be used (Puonti et al., 2013). Khan et al. (2008) propose to use the segmentation output from the method by Fischl et al. (2002) to drive registration of a secondary manually labelled template.

The information fusion approach described by Barra and Boire (2001) segments structures based on a model of expert knowledge. General descriptions of experts of segmentation criteria are converted to image-based pieces of information, from which a fusion process finds the final segmentation. Fuzzy neural networks have also been proposed for segmentation (Jian et al., 2002). An approach based on discriminative dictionary learning was proposed by Benkarim et al. (2014).

Finally, a number of methods segment structures by fitting a mesh to the imaging data. The method described by Belitz and Rohr (2006) uses an active surface model (ASM), which is based on image-based edge detection. Asl and Soltanian-Zadeh (2008) also use a shape model, but optimise with respect to the image entropy within structures instead. Liu et al. (2007) propose a method that incorporates a probabilistic atlas and Uzunbas et al. (2010) and Cerrolaza et al. (2012) take advantage of the spatial relationships between structures. FIRST (Patenaude et al., 2011) uses a Bayesian appearance model that links the intensities around a deformable shape to the spatial configuration of the shape. The intensities and possible shape variations are derived from a set of manually labelled training segmentations.

All of the methods described above segment subcortical structures on the basis of only a *T*_1_-weighted scan. In the case of a poorly visible structure like the globus pallidus, a different contrast, like a *T*_2_-weighted scan, can be more useful. Magnotta et al. (1999) propose a method that uses multiple contrasts to segment the brain tissue into grey matter (GM), white matter (WM) and cerebrospinal fluid (CSF). An artificial neural network is then trained using the tissue-type maps and a set of manual labels to label the subcortical structures. Multi-modal extensions have also been proposed to atlas-based methods (Xiao et al., 2012) and random-field-based segmentation (Marrakchi-Kacem et al., 2010, related to Poupon et al., 1998). Kim et al. (2014) propose a method that combines explicit edge detection on multiple modalities with a statistical shape model that is trained using multiple manual segmentations.

Many of the methods described above need to be trained using manually labelled data. The required amount of training data can be either a single segmentation for deterministic atlas-based methods or a larger set of labelled scans for the classifier and probabilistic atlas-based methods. The degree to which methods are constrained to the properties of these training segmentations varies. Fully atlas-based methods will not capture details at a higher resolution than that of the deformation field, while the random-field-based approach in FreeSurfer should allow it to detect smaller variations more flexibly. FIRST parameterises shapes using a limited number of modes of variation that are derived from the training segmentations.

If segmentation is constrained by the variations that were seen in the training data, this may have undesirable consequences when applying the method to populations that are different from the training set. An example would be patient groups, where anatomy might be significantly different from the normal population. Segmentation results in such a group would be biased towards the appearance of the structure in the training data. The same holds for the less typical cases within the normal population; these might not be within the variability observed in the training group. In the case of pathology, there might also be unique variations in single subjects.

Because of these potential differences compared to the training group, it is a desirable property of a segmentation method to require only a limited number of manual segmentations, as this makes retraining the method for a new population more tractable. The segmentation method could then be re-trained to the images that are to be segmented, without requiring a full set of manually labelled training segmentations. This would remove much of the bias towards the original training group, but would not solve the more idiosyncratic cases. To address this problem, a more flexible approach with weaker constraints is required. A further potential disadvantage of using manually labelled training segmentations is that it introduces some subjectivity in the segmentation process. We would like a new method to be more data-driven and less reliant on manual segmentations. When a method can be more easily retrained, it can also be adapted if the image contrast in a study is fundamentally different from that in the training data. This might be the case if acquisition is optimised for the segmentation of a particular structure, for example the globus pallidus which is hard to segment on a *T*_1_-weighted image but much clearer on a *T*_2_-weighted one.

In this paper, we describe a multimodal segmentation method that can simultaneously use information in multiple volumes with different contrasts. This has two potential advantages. First, if not all boundaries of a structure are visible in each contrast, it allows the method to combine the complementary information. Secondly, in cases of low contrast, the availability of multiple images can help segmentation as it effectively increases the contrast-to-noise ratio. The method detects edges in images based on their intensity profiles, allowing it to capture shape variations that did not occur in the training set. We use a generative model for the intensity profiles perpendicular to a deformable mesh, somewhat like Patenaude et al. (2011), but unlike that method it can learn in an unsupervised fashion from unlabelled training data. The user input is limited to the reference mesh, which may be derived from a single manual segmentation or from an existing atlas, and a set of priors describing the general form of contrast changes at the boundaries of the structure. We describe an automatic procedure for setting these parameters for the striatum and globus pallidus, to enable retraining on different datasets and populations. To use the method with new contrasts and structures, the user can specify the priors by hand using the same principles described in the paper. We will refer to the method as ‘multimodal image segmentation tool (MIST)’.

## Methods

We aim to integrate the information in multiple imaging modalities to produce accurate segmentations of subcortical structures. As a starting point, we take a reference mesh that roughly corresponds to the structure that we are interested in and map this to a subject's data using nonlinear registration. The reference mesh may, for example, be derived from an atlas. The method should then deform the mesh to optimally align with the physical boundaries of the structure of interest. The intensity profiles along the normal vectors at the vertices of the reference mesh are used to estimate the adjustments that are needed to make the reference mesh align with the anatomy.

An important question is how the method will be able to relate observed image intensities to the actual structural boundaries. If we had used manually labelled training segmentations, this would be trivial, as the physical boundary would be defined as the point where the person that labelled the image drew the boundary. However, as we do not have such manual segmentations, we need to devise an alternative method. The approach that we take is based on the simultaneous alignment of the profiles observed in an unlabelled training group, which is combined with a prior that specifies, in general terms, what intensity changes we expect near the boundary. Such a prior resolves the ambiguity in the connection between an edge in the observed intensity profiles and the physical boundary.

The framework that we will use is a probabilistic one, where a generative model describes how between-subject anatomical variability gives rise to the observed intensities. We will use a Bayesian approach where we find estimates of the model parameters in a training phase. The trained model is then used to obtain an approximate maximum a posteriori (MAP) estimate of the deformations needed to segment a structure in a new set of images.

In the next sections, an overview of the generative model will be given first, followed by a discussion of how we obtain estimates of its parameters and how the priors need to be set. More practical issues, such as prerequisite image registration, are discussed in the final part of this section. We will assume for now that the reference mesh is roughly aligned with the images that are to be segmented.

### Generative model

The model that we use can be seen as consisting of two parts: A part that we will call the *shape model*, which describes how the displacements at different vertices vary and covary, and a second part, the *intensity model*, which is independent for each vertex and which describes how the intensities arise given a displacement.

#### Shape model

We assume that the displacements along the vertex normals are sampled from a multivariate normal (MVN) distribution with mean *μ*^*s*^ and precision **Λ**^*s*^:

(1) $$p\left(\left(\delta \right|\mu^{s},\Lambda^{s}\right)=N_{N}\left(\left(\delta \right|\mu^{s},\Lambda^{s}\right)\text{.}$$

In this equation, *δ* is the *N*-dimensional vector consisting of the displacements of all *N* vertices along the normals.

#### Priors for the shape model

The role of the shape model is to learn the differences between the mean location of the structural boundary in the training data and the reference mesh. It also constrains the segmentation by controlling the covariance between vertices. We use a Normal–Wishart distribution as the prior for the mean and precision of the shape model:

(2) $${Nw}_{k}\left(\mu^{s},\Lambda^{s}\right)=N_{k}\left(\left(\mu^{s}\right|\mu_{0}^{s},n_{0}\Lambda^{s}\right){Wi}_{k}\left(\left(\Lambda^{s}\right|\alpha^{s},\beta_{s}\right)\text{.}$$

The prior mean *μ*_0_^*s*^ is set to zero and the elements of the covariance (inverse precision) prior are specified as a Gaussian function of the surface distances between the vertices:

(3) $$\beta_{ij}^{s}=h^{2}e^{-\frac{d_{ij}^{2}}{2w^{2}}}\text{,}$$

where *d*_*ij*_ is the number of edges between vertices *i* and *j*, *w* is the width parameter and *h*^2^ is the expected on-diagonal value of the covariance matrix; we will refer to *h* as the height parameter. The width parameter controls how strong we expect the correlation between neighbouring vertices to be. The parameters *n*_0_^*s*^ and *α*^*s*^ control the width of the prior distributions. More details about all of these parameters are given in Appendix A.

#### Intensity model

In order to be able to later fit the mesh to imaging data, we need to define the relationship between the two. In this section we shall only be concerned with a single vertex on the mesh, as the form of the model is identical and independent for all vertices. We start with a simple model where the intensities sampled at equidistant points along the normals of the anatomical structure are represented by an MVN distribution:

(4) $$p\left(\left(y\right|\mu ,\Lambda \right)=N_{k}\left(\left(y\right|\mu ,\Lambda \right)\text{,}$$

where **y** is the *k*-dimensional vector of the intensities that make up the profile and *μ* and **Λ** are the mean and precision. The profile **y** is centred around the physical boundary of the structure.

To be able to estimate displacements, the profile that is considered in the image is shorter than the reference profile *μ*. The observed profile **y**′ has length *k*′ and is centred around the reference shape. We assume that this is sampled from the distribution described by Eq. (4). As the observed profile is shorter than *μ*, however, we need to marginalise over the unobserved points. For an MVN distribution, this is as simple as dropping the corresponding dimensions. The number of dimensions that need to be dropped on either end of the profile is determined by the integer displacement $\underline{\delta }$ at this vertex:

(5) $$p\left(y^{'}\left|\underline{\delta }=\left\lfloor \delta -\delta_{\text{min}}+\frac{1}{2}\right\rfloor ,\mu ,\Lambda \right)\right)=\int \ldots \int p\left(y\left|\mu ,\Lambda \right)\right)dy^{0}\ldots dy^{\underline{\delta }-1}dy^{\underline{\delta }+k^{'}}\ldots dy^{k}$$

(6) $$=N_{k'}\left(\left(y^{\prime }\right|\mu^{\delta },\Lambda^{\delta }\right)\text{,}$$

with the integer displacement $\underline{\delta }$ defined with respect to the maximum negative displacement. The maximum integer displacement is Δ = *k* − *k*′. In practice, we will use the same value for Δ and *k*′, which means that, for example, an observed profile of 20 points would have maximum displacements of 10 points inwards and 10 points outwards. The length of the reference profile would be *k* = 40 in this case. We will use the superscript *δ* to denote taking the subvector or submatrix corresponding to an integer displacement $\underline{\delta }$. It is useful to note at this point that in the training stage, due to the lack of manually labelled training data, the displacement *δ* will be unknown. As we will see later, this ambiguity can be resolved by using informative priors.

The anatomy that is observed along the normal at a given vertex may not be the same for all subjects. Examples of this might be vessels, whose exact location varies with respect to the vertices, or the exact points where neighbouring structures start to be connected to the structure of interest. In order to model such variability, we extend the simple model in Eq. (5) by replacing the single MVN by a mixture model with *N*_*r*_ components:

(7) $$p\left(y\left|\underline{\delta },\mu_{0},\Lambda_{0},\ldots ,\mu_{N_{r}},\Lambda_{N_{r}},\theta \right)\right)=\sum_{r=1}^{N_{r}}p\left(y^{'}\left|\underline{\delta },\mu_{r},\Lambda_{r}\right)\right)p\left(r\left|\theta \right)\right)$$

(8) $$=\sum_{r=1}^{N_{r}}N_{k^{\prime }}\left(y_{r}^{\prime }\left|\mu_{r}^{\delta },\Lambda_{r}^{\delta }\right)\right)\mathrm{Cat}\left(r\left|\theta \right)\right)\text{,}$$

where Cat(*r*|*θ*) is the categorical distribution and where *μ*_*r*_ and **Λ**_*r*_ are the mean and precision matrix for component *r*.

The final extension that we need to make to the profile model is the extension to multiple modalities. This is relatively straightforward:

(9) $$p\left(y_{0},\ldots ,y_{N_{m}}\left|\underline{\delta }\right),M,L,\theta \right)=\prod_{m=1}^{N_{m}}\sum_{r=1}^{N_{r}}N_{k^{'}}\left(y_{m}\left|\mu_{mr}^{\delta },\Lambda_{mr}^{\delta }\right)\right)\mathrm{Cat}\left(r\left|\theta_{m}\right)\right)\text{,}$$

where *N*_*m*_ is the number of modalities and where we have used the shorthands *M*, *L* and *θ* to denote all mean vectors *μ*_*mr*_, all mean precision matrices **Λ**_*mr*_ and all mixing parameter vectors *θ*_*m*_, respectively.

#### Parameterisation of the covariance matrix

The precision matrices in the intensity model are parameterised through the inverse of the covariance matrix:

(10) $${\left(\Lambda_{mr}\right)}^{-1}=\Sigma_{mr}={GD}_{mr}G\text{,}$$

where D_*mr*_ is the diagonal matrix that parameterises the covariance matrix and G is a symmetric matrix that determines the smoothness. When fitting the model, G is a constant parameter, while D_*mr*_ needs to be determined from the data. Both Σ and the precision matrix Λ = (Σ)^− 1^ need to be computed frequently and this can be done quickly as G^− 1^ is constant and needs to be computed only once and D_*mr*_, being diagonal, is trivial to invert. The elements of the matrix G are:

(11) $$G_{ij}=e^{-\frac{{\left(i-j\right)}^{2}}{2\sigma^{I}}}\text{,}$$

where *σ*^*I*^ is the smoothness parameter of the intensity model.

#### Priors for the intensity model

The priors that we will set on *μ*_*mr*_ and **Λ**_*mr*_ are crucial for the method to be able to detect edges rather than to rely on manual segmentations to build the reference profiles. Without prior information, the relationship between the physical boundary and the mean profile intensity is undefined. By setting a prior that specifies what we expect an edge to look like, this ambiguity is resolved. We use a Normal–Wishart prior for *p*(*μ*_*mr*_, **Λ**_*mr*_):

(12) $${Nw}_{k}\left(\mu_{mr},\Lambda_{mr}\right)=N_{k}\left(\left(\mu_{mr}\right|\mu_{mr}^{0},n_{0}\Lambda_{mr}\right){Wi}_{k}\left(\left(\Lambda_{mr}\right|\alpha^{0},\beta_{mr}^{0}\right)$$

where Wi_*k*_ denotes a Wishart distribution. The hyperparameters that we need to set are the parameters *μ*_*mr*_^0^, *n*_0_, *α*^0^ and *β*_*mr*_^0^. The parameters *μ*_*mr*_^0^ and *n*_0_ control the mean and variance of the distribution on *μ*_*mr*_ and the parameters *β*_*mr*_^0^ and *α*^0^ control the mean and variance of **Λ**_*mr*_.

In general, when setting up the method to segment a structure, we will have some general idea about what the intensity profiles perpendicular to the structural boundaries look like. We would like to specify these ideas once for the structure (i.e. we want to use the same priors for all vertices) and therefore the specification needs to be relatively general. The model can then learn more specific features of the profile and the probabilities of the mixture components appearing at a specific vertex. We will parameterise the prior profiles by selecting one of the functions in Table 1.

We have parameterised the covariance matrices through Eq. (10) and we will use a similar structure for the covariance hyperparameter *β*_*mr*_^0^ of the Normal–Wishart distribution. The Wishart distribution includes covariance matrices that cannot be generated through Eq. (10) and the parameterisation of (**Λ**_*mr*_)^−1^ effectively acts as an additional constraint to enforce the desired off-diagonal structure. While in principle different values can be specified for different points along the profile, there is little advantage to doing so and we use a scalar value *β*_*mr*_ for all of the coefficients:

(13) $$\beta_{mr}^{0}=\beta_{mr}GG\text{.}$$

For the mixing parameters *θ*_*m*_, we use a Dirichlet prior Dir(*θ*_*m*_|*α*). All components of the hyperparameter *α* are set to the same value. This value determines a preference towards either equal mixing or selection of a single component.

#### Full model

Combining Eqs. (1), (9) yields the joint distribution for the full generative model:

(14) $$\begin{array}{l} p\left(\left(Y_{1},\ldots ,Y_{N},\delta \right|M_{1},L_{1},\theta_{1},\ldots ,M_{N},L_{N},\theta_{N},\mu^{s},\Lambda^{s}\right)\\ =N_{m}\left(\delta \left|\mu^{s},\Lambda^{s}\right)\right)\prod_{i=1}^{N}\prod_{m=1}^{N_{m}}\sum_{r=1}^{N_{r}}N_{k^{\prime }}\left(y_{im}\left|\mu_{imr}^{\delta_{i}},\Lambda_{imr}^{\delta_{i}}\right)\right)\mathrm{Cat}\left(r\left|\theta_{im}\right)\right)\text{,} \end{array}$$

where *N* is the number of vertices and where *Y*_*i*_ denotes all vectors **y**_*im*_. The subscripts *δ*_*i*_ refer to the integer displacements corresponding to the components of *δ*. Eq. (14) will only be evaluated for these integer values.

Eq. (14) shows that the individual profile mixture models at different vertices are thus linked through the shape distribution N_*m*_(*δ*|*μ*^*s*^, **Λ**^*s*^). The model parameters will be learned in the training stage, which will be described below. The training stage makes use of the priors introduced above to find posterior estimates of the parameters. Due to the complexity of the model, in practice we will train the profile and shape parts of the model sequentially. For the shape model, we will use the analytical conjugate process and this part of the model is fully Bayesian. Maximum a posteriori (MAP) parameter estimates are used in the intensity model.

### Model estimation

The intensity models at different vertices are in principle linked through the shape model. Estimating the full model is computationally intensive, however, given the large number of vertices. For this reason, we estimate the intensity models for all vertices independently and use point estimates for their parameters when estimating the shape model.

#### Estimating *μ*_*mr*_, **Λ**_*mr*_ and *θ*_*m*_ from training data

The parameters *μ*_*mr*_, **Λ**_*mr*_ and *θ*_*m*_ are unknown and we want to learn these from a set of training data. From Eq. (9), it is easily seen that, for a single vertex, the combined probability for the full set of intensity profiles **z**_*sm*_ in the training data is

(15) $$p\left(z_{1},\ldots ,z_{S}\left|\delta_{1},\ldots ,\delta_{S},M,L,\theta \right)\right)=\prod_{s=1}^{N_{t}}\prod_{m=1}^{N_{m}}\sum_{r=1}^{N_{r}}N_{k^{'}}\left(z_{sm}\left|\mu_{mr}^{\delta_{s}},\Lambda_{mr}^{\delta_{s}}\right)\right)\mathrm{Cat}\left(r\left|\theta_{m}\right)\right)\text{,}$$

where *δ*_*s*_ is the displacement for subject *s*, *N*_*t*_ is the number of training subjects and the vertex index *i* has been omitted. Applying Bayes' rule yields

(16) $$\begin{array}{l} p\left(M,L,\theta ,\delta_{1},\ldots ,\delta_{S}\left|z_{1},\ldots ,z_{S}\right)\right)\\ \propto p\left(z_{1},\ldots ,z_{S}\left|M,L,\theta ,\delta_{1},\ldots ,\delta_{S}\right)\right)\left(\prod_{m=1}^{N_{m}}\prod_{r=1}^{N_{r}}p\left(\mu_{mr},\Lambda_{mr}\right)\right)\left(\prod_{s=1}^{N_{t}}p\left(\delta_{s}\right)\right)p\left(\theta \right)\\ =\left(\prod_{m=1}^{N_{m}}Dir\left(\theta_{m}\left|\alpha \right)\right)\right)\left(\prod_{m=1}^{N_{m}}\prod_{r=1}^{N_{r}}Nw_{k}\left(\mu_{mr},\Lambda_{mr}\left|\mu_{mr}^{0},n_{0}\left|\alpha^{0},\beta_{mr}\right)\right)\right)\right)\\ \times \overset{S}{\underset{s=1}{∐}}N\left(\delta_{s}\left|\mu_{d},\lambda_{d}\right)\right)\prod_{m=1}^{N_{m}}\sum_{r=1}^{N_{r}}N_{k^{\prime }}\left(z_{sm}\left|\mu_{mr}^{\delta_{s}},\Lambda_{mr}^{\delta_{s}}\right)\right)\mathrm{Cat}\left(r\left|\theta_{m}\right)\right)\text{.} \end{array}$$

The priors N(*δ*_*s*_|*μ*_*δ*_, *λ*_*δ*_) replace the shape model. This replacement is necessary to be able to train the profile part of the model separately for each vertex, but it is an approximation to the full model. The parameter *μ*_*δ*_ is set to zero, i.e. the distribution is centred around the reference shape.

When fitting the full model to new data, we will use point estimates for the parameters *μ*_*mr*_, **Λ**_*mr*_ and *θ*_*m*_. These are the MAP estimates in the single-vertex isolated intensity model. To simplify the problem of finding these, it is useful to first marginalise out *δ*_1_, …, *δ*_*S*_:

(17) $$\begin{array}{l} p\left(M,L,\theta \left|z_{1},\ldots ,z_{S}\right)\right)\propto p\left(\theta \right)\left(\prod_{m=1}^{N_{m}}\prod_{r=1}^{N_{r}}p\left(\mu_{mr},\Lambda_{mr}\right)\right)\\ \times \prod_{s=1}^{S}\sum_{\delta_{s}=0}^{\Delta }p\left(\delta_{s}\right)\prod_{m=1}^{N_{m}}\sum_{r=1}^{N_{r}}N_{k^{'}}\left(z_{sm}\left|\mu_{mr}^{\delta_{s}},\Lambda_{mr}^{\delta_{s}}\right)\right)\mathrm{Cat}\left(r\left|\theta_{m}\right)\right)\text{,} \end{array}$$

where the sum is over the integer values of *δ*_*s*_. The MAP estimates of the parameters *μ*_*mr*_, **Λ**_*mr*_ and *θ*_*m*_ are the values where this function is at its global maximum. For **Λ**_*mr*_ the parameterisation that was described above is used.

To perform the optimisation, the method of moving asymptotes (MMA, Svanberg, 2002) is used, as implemented in the NLopt optimisation library (Johnson, http://ab-initio.mit.edu/nlopt). This algorithm was chosen because it converged more quickly in practice than the alternatives that were considered. Optimisation is performed using the logarithm of the probability given by Eq. (17). The logarithm of the probability and its derivatives, which are needed for MMA, are given in the Supplementary material.

#### Training the shape model

As we train the intensity models in isolation, we can take the most likely displacements for all subjects and use these to train the shape part of the model. We use the standard training process using conjugate priors; the details are given in the Supplementary material.

#### Estimating the approximate full model

With the trained intensity models and the shape model we can obtain an estimate of the vector of displacements *δ* for a new set of data given the training data. It is not difficult to see that the conditional probability of the continuous displacements *δ* is proportional to Eq. (14):

(18) $$\begin{array}{l} p\left(\delta \left|Y_{1},\ldots ,Y_{N},J,\delta_{0}^{z},\ldots ,\delta_{N_{t}}^{z}\right)\right)=\frac{p\left(Y_{1},\ldots ,Y_{N},\delta \left|J,\delta_{0}^{z},\ldots ,\delta_{N_{t}}^{z}\right)\right)}{\int p\left(Y_{1},\ldots ,Y_{N},\delta \left|J,\delta_{0}^{z},\ldots ,\delta_{N_{t}}^{z}\right)\right)d\delta_{1}\ldots d\delta_{N}}\\ \;\propto p\left(Y_{1},\ldots ,Y_{N},\delta \left|J\right)\right)p\left(\delta |\delta_{0}^{z},\ldots ,\delta_{N_{t}}^{z}\right)\text{,} \end{array}$$

where *J* = {*M*_1_, *L*_1_, *θ*_1_, …, *M*_*N*_, *L*_*N*_, *θ*_*N*_} denotes the parameters of the intensity models at all vertices. As mentioned before, this depends on *M*_*i*_, *L*_*i*_ and *θ*_*i*_ explicitly, as we use MAP point estimates from the training stage when fitting to new data. The final segmentation is given by the vector *δ* for which this distribution has its maximum. The details of the optimisation procedure are given in the Supplementary material.

### Parameters

The most important parameters to be set up before training the model are the shapes and intensities of the prior mean profiles. As will be seen below, the shapes, i.e. the selection of a function from Table 1 along with the specification of any shape parameters, depends mostly on anatomy, whereas good choices for the intensities can be derived from the training data themselves in an automated fashion.

**Fig. 1:**
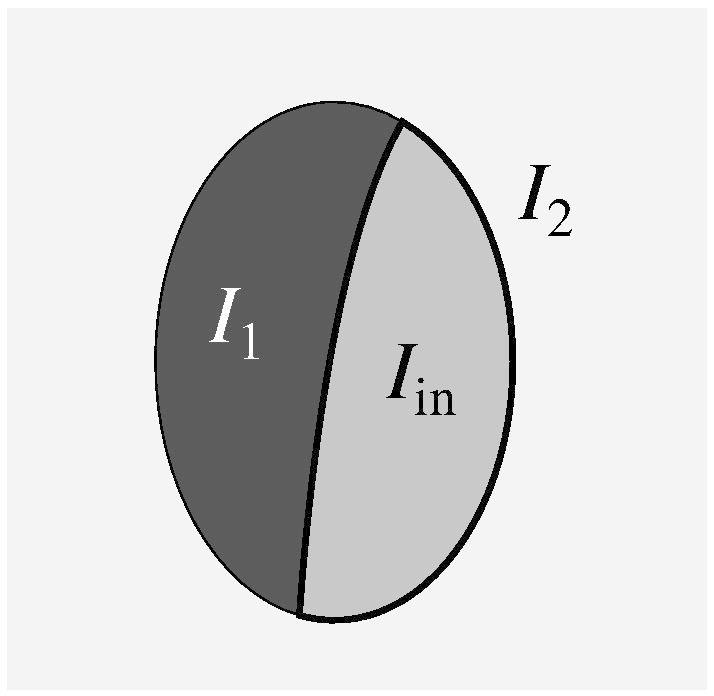
Different intensities (white and dark grey) around the structure to be segmented (light grey).

While there are a significant number of parameters to be specified in setting up the priors, the meaning of these parameters is relatively transparent. The problem's geometry is illustrated by Fig. 1, where the light grey shape represents the structure to be segmented. For purposes of specifying the prior, it is assumed that image intensity *I*_in_ inside the structure is close to homogeneous. In this example, the structure of interest has two neighbouring structures with intensities *I*_1_ and *I*_2_. These intensities are highly acquisition-dependent, but sufficiently good estimates can be found by automatically determining the intensities inside atlas-derived volumetric masks in the training dataset.

In addition to specifying the intensities, suitable functions need to be selected from Table 1. These need to have some resemblance to the profiles actually encountered in the training data, as their role is to determine what point on the intensity profile corresponds to the physical boundary. In cases where the outside intensity is homogeneous in a region that extends beyond the profile length considered (e.g. *I*_2_), a step function is a good choice, but in other cases, for example where there is a thin sheet of white matter, an exponential prior may be more appropriate as this forces the boundary to be adjacent to the sheet; a step function would leave the location within the relevant half ambiguous.

In practice, we use a Python script to automatically set up the hyperparameters in a rule-based fashion. This script allows a user of the method to automatically retrain it for any dataset that has similar modalities to those used in this paper. The script uses the median intensities in a number of predefined regions in the user's images to automatically set the intensity parameters of the priors. A full description of how the priors and other parameters are set up is given in Appendix A. An automated procedure to set the parameters in order to retrain the method on a new dataset is also explained there.

In order to be able to directly compare uni-modal and multi-modal segmentation accuracy with the same method, we also trained the model on only the *T*_1_-weighted data with the same parameters that were used for the *T*_1_-weighted modality in the multimodal case.

### Registration and intensity normalisation

The approach we take in segmenting a structure is to first register the brain to a standard space template volumetrically. The first aim of this step is to remove the pose of the head and any global scaling, which is achieved by the affine registration step. A nonlinear registration step is also run to remove as much between-subject anatomical variability as possible. Note that this is merely to reduce the size of the variations that need to be explained by the shape model of the segmentation method; the final segmentation output will still be in the undistorted subject-native coordinates.

Registration is performed using the FLIRT and FNIRT tools in FSL (Jenkinson et al., 2002, Andersson et al., 2010). The *T*_1_-weighted volume is registered to the 2 mm isotropic resolution version of the MNI152 template. Where needed, the other modalities are then registered to the *T*_1_ weighted volume using mutual information or, in the case of the diffusion data, using boundary-based registration of the fractional anisotropy (FA) image (Greve and Fischl, 2009). In the 7 T dataset, which will be described below, all modalities were registered to the FLASH volumes instead.

Before fitting the model, the intensity of all volumes for each modality is scaled automatically to have the same mean intensity across subjects in a bounding box around the structure to be segmented. For structures like the caudate nucleus, the amount of cerebrospinal fluid (CSF) inside such a box can vary substantially from subject to subject. In many contrasts, CSF exhibits either very high or very low intensities and as this might interfere with normalisation, CSF voxels were not included in the calculation of the mean for any of the structures. FAST (Zhang et al., 2001) was used to create the CSF mask. Normalisation was not used for the FA volumes (see below) and for the QSM volumes (see below) an additive normalisation procedure was used instead in order to deal with negative values correctly.

### Mesh generation

The reference meshes for our segmentation method were generated from the probabilistic Harvard–Oxford subcortical atlas (supplied with FSL) using the marching cubes algorithm as implemented in VTK (http://www.vtk.org/). The 2 mm isotropic resolution version of the atlas was used, meaning that points on the meshes are approximately 2 mm apart. For the putamen and globus pallidus, the threshold was set at 50%. For the caudate nucleus and nucleus accumbens simple thresholding did not produce a suitable mesh, as the tail of the caudate has relatively low atlas probabilities due to anatomical variation. To compensate for this, voxels were upweighted based on their Euclidean distance from the highest-probability region of the structure. A regularisation and smoothing step implemented in FIRST (Patenaude et al., 2011) was applied to all meshes to produce more regular triangles and smoother surfaces.

We chose to use a single mesh representing the caudate nucleus and nucleus accumbens joined together, because there is little to no visible structure dividing the two in the images we are using. The merged structure was created by taking the sum of the probabilistic maps for the two structures before generating the mesh.

### Datasets

The three datasets described below were used to evaluate the method.

#### Human Connectome Project (HCP80) dataset

The first is the 80 unrelated subjects subset of the Human Connectome Project (HCP80) dataset. All subjects are healthy adults. The following scans were acquired on a Siemens Skyra 3 T system with a 32-channel head coil (Van Essen et al., 2012, Van Essen et al., 2013):

• The average of two 0.7 mm isotropic resolution *T*_1_-weighted MPRAGE acquisitions with a 320 × 320 × 256 imaging matrix, repetition time (TR) = 2400 ms, echo time (TE) = 2.14 ms, inversion time (TI) = 1000 ms, flip angle = 8° and acceleration factor 2. The acquisition time for each scan was 7:40.
• The average of two 0.7 mm isotropic resolution *T*_2_-weighted 3D-SPACE acquisitions with a 320 × 320 × 256 imaging matrix, TR = 3200 ms, TE = 565 ms, variable flip angle and acceleration factor 2. The acquisition time for each scan was 8:24.
• An extensive diffusion-weighted imaging (DWI) acquisition with 1.25 mm isotropic resolution, multiband echo-planar imaging (EPI) with a 168 × 168 imaging matrix, 111 slices, TR = 5520 ms, TE = 89.5 ms, 6/8 phase partial fourier and multiband factor 3 (Moeller et al., 2010, Feinberg et al., 2010, Setsompop et al., 2012, Sotiropoulos et al., 2013). All volumes were acquired twice with left–right and right–left phase encoding polarities. The acquisition time was approximately one hour. Only the 90 directions with diffusion weighting *b* = 1000 s/mm^2^ and 18 volumes with *b* = 0 were used for calculating the FA images.

All of these images were corrected for gradient nonlinearity induced distortions by the HCP pipeline (Glasser et al., 2013). The pipeline additionally corrected the intensities of the *T*_1_- and *T*_2_-weighted images for *B*_1_ inhomogeneities. Geometric distortions due to *B*_0_ inhomogeneity and eddy currents were corrected by the pipeline using EDDY and TOPUP, making use of the additional images with reversed phase encoding (Andersson et al., 2003, Andersson et al., 2012) Fractional anisotropy (FA) images were calculated using FSL's DTIFIT.

The HCP80 dataset contains 77 usable subjects, 10 of which were used for experimentation and 10 were manually labelled for the quantitative evaluation of our method. This left a training group of 57 subjects. All results shown in this paper were obtained by training the model on this training group and segmenting the subjects in the labelled group.

#### 7 T dataset

The second set of structural data were the young subjects of the publicly available dataset described by Forstmann et al. (2014). These data were acquired using a 7 T Siemens Magnetom MRI using a 24-channel head array Nova coil (NOVA Medical Inc., Wilmington MA):

• A *T*_1_-weighted MP2RAGE slab (Marques et al., 2010) which consisted of 128 slices with an acquisition time of 9:07 min (TR = 5000 ms; TE = 3.71 ms; TI_1_/TI_2_ = 900/2750 ms; flip angle = 5°/3°; bandwidth = 240 Hz/Px; voxel size = 0.6 mm isotropic).
• A *T*_1_-weighted whole brain MP2RAGE acquisition which had 240 sagittal slices with an acquisition time of 10:57 min (repetition time (TR) = 5000 ms; echo time (TE) = 2.45 ms; inversion times TI_1_/TI_2_ = 900/2750 ms; flip angle = 5°/3°; bandwidth = 250 Hz/Px; voxel size = 0.7 mm isotropic)
• A *T*_2_^⁎^-weighted 3D FLASH acquisition. The FLASH slab (Haase et al., 1986) consisted of 128 slices with an acquisition time of 17:18 min (TR = 41 ms and three different echo times (TE): 11.22/20.39/29.57 ms; flip angle = 14°; bandwidth = 160 Hz/Px; voxel size = 0.5 mm isotropic).

The imaging slab for the limited field-of-view acquisition was 64 mm thick for the FLASH protocol and 76.8 mm for the MP2RAGE protocol and positioned to capture the subcortical structures. Both slab sequences consisted of axial slices tilted − 23° to the true axial plane in scanner coordinates. The QSM volume was calculated using the phase information of the FLASH MRI sequence and the method proposed by (Schweser et al., 2013).

The 7 T dataset consisted of 29 usable subjects; one subject was not used because registration of the whole-brain MP2RAGE volume to the FLASH slab failed. Fourteen of these subjects were male and 15 were female. Their mean age was 23.8 years with a standard deviation of 2.3 years. For quantitative evaluation, a ‘leave 5 out’ scheme was used, where per block of 5 or 4 subjects the method was trained on the other 24 or 25 subjects.

#### Clinical HD dataset

To investigate how well the method performs on data acquired using more typical protocols and in the presence of pathology, it was also applied to the dataset from a Huntington's disease (HD) study. HD is an informative test case as the disease is associated with severe atrophy of subcortical grey matter. These data and the manual segmentations are described in detail in the original papers (Douaud et al., 2006, Douaud et al., 2009) and acquired as part of the MIG-HD (Multicentric Intracerebral Grafting in HD) project.

The dataset consists of ten patients and six control subjects from the original study. The number of control subjects included was limited to six, as this was the only consistent subset of the control data that had both identical acquisition protocols for the structural images and for which diffusion data were available. For each subject the following scans were obtained on a General Electric 1.5 T Signa system with a birdcage head coil with 40 mT/m maximum gradient strength:

• A *T*_1_-weighted scan using a 3D inversion recovery fast spoiled gradient recalled (IR-FSPGR) acquisition with 0.9375 mm × 0.9375 mm × 1.2 mm resolution, matrix size 256 × 256 × 124, TI = 620 ms, TE = 2 ms, TE = 20.3 ms, flip angle = 10° and bandwidth = 7.81 kHz.
• Diffusion-weighted echo-planar imaging (EPI) with matrix size 128 × 128, reconstructed as 256 × 256 to yield 0.9375 × 0.9375 in-plane resolution with 2 mm slice thickness, 60 slices, TE = 71 ms, TR = 2500 ms, flip angle = 90°, bandwidth = 125 kHz, 41 diffusion directions, *b* = 700 s/mm^2^ and one volume with *b* = 0.

### Manual segmentations

#### 7 T dataset

The manual segmentations in the 7 T data were described in Keuken et al. (2014). The putamen, caudate nucleus and nucleus accumbens, along with the islands of grey matter between the putamen and the caudate nucleus and nucleus accumbens, were segmented as a single structure. Because all segmentation methods evaluated in this paper segment the different parts of the striatum separately and none segment the grey matter islands, these islands were removed from the segmentation masks. The internal and external parts of the globus pallidus were labelled using the QSM volumes. As there is a gap between the parts, voxels for which the sum of the Euclidean distances to the internal and the to the external part was smaller than 2.5 mm were added to the combined mask. This effectively closes the gap between the two parts.

#### HCP80 dataset

Manual segmentation of the striatum in the HCP80 dataset was performed by a single rater using the *T*_1_-weighted volumes and the same guidelines as used in Keuken et al. (2014). The globus pallidus was not segmented as this dataset did not include QSM images.

#### HD dataset

Manual segmentation of the putamen, caudate nucleus and ventral striatum in the HD dataset was described in (Douaud et al., 2006). The scans were resliced to have the anterior commissure (AC) and posterior commissure (PC) in the same axial plane. This plane was used as the lower boundary of the globus pallidus in the manual segmentations. In the comparisons between methods for the HD dataset, the automatic segmentations were also cut off in the AC–PC plane to be consistent with the manual segmentation protocol.

### Methods comparison

The segmentations produced by MIST, FIRST (Patenaude et al., 2011) and FreeSurfer (Fischl et al., 2002) were quantitatively compared with manual segmentations in all three datasets (HCP80, 7 T and HD). To compare the methods, three different comparisons were performed. The first of these compares the Dice scores between different methods.^1^ The second comparison looks at the mean distance between the meshes produced by the different methods. Finally, the third comparison compares the correlation coefficients between the automatically segmented mask volumes and manual segmentations. The general procedure used for this comparison is described here, as well as a number of processing steps that are specific to one or two of the datasets.

The mesh-based output from MIST was converted to voxel-based masks in order to be able to compare to the manual segmentation. This was achieved by testing for all voxels' vertices whether they were inside the mesh. A voxel was considered to be inside the mesh if at least one of its vertices was inside. The vtkSelectEnclosedPoints filter in VTK was used for this procedure.

In order to be able to compute the average distances between meshes, the manual segmentations and FreeSurfer segmentations were converted to meshes using the vtkMarchingCubes filter. Where meshes needed to be combined for the comparison, such as for the caudate and accumbens meshes from FIRST, only points that are at least 2 mm from the other mesh were used in the distance calculation to ensure that no internal boundaries were present.

In the HCP80 and 7 T datasets, results were compared to those obtained using FIRST with and without its boundary correction post-processing step. This step uses a voxel-based mixture model to reassess which voxels on the boundary of a structure should and which should not be part of the structure. In the 7 T dataset, the segmentations also needed to be registered to the scans on which the manual segmentations were performed: The partial FOV MP2RAGE scans for striatum and the QSM volume for the globus pallidus. All segmentation methods subdivide the striatum into multiple structures; the masks for the putamen, caudate nucleus and nucleus accumbens were combined into a single striatum mask.

In the HCP80 dataset, the FreeSurfer segmentations that are provided with the pre-processed structural datasets by the Human Connectome Project were used. FIRST was run with its default options on the *T*_1_-weighted volume. The segmentations produced by multi-modal segmentation were also output in the coordinates of this volume.

In the 7 T dataset, FreeSurfer was run on the brain-extracted whole-brain MP2RAGE volume as whole brain coverage is a requirement of its processing pipeline. The segmentation masks were linearly upsampled to match the full resolution MP2RAGE volume and registered to both the partial FOV MP2RAGE and FLASH volumes as the manual segmentations were performed on these images. FIRST was run on both the whole-brain and partial FOV MP2RAGE volumes and the resulting segmentations were registered with the partial FOV MP2RAGE and FLASH images. In the segmentations using MIST, the partial FOV MP2RAGE volumes were used.

## Results

### Automatic edge alignment

**Fig. 2:**
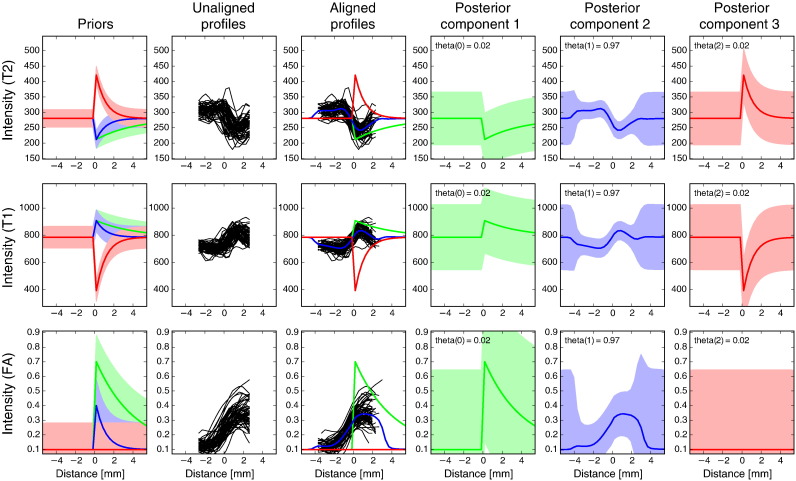
Profile model for a single vertex on the inferior boundary of the putamen. First column: specified priors (green: first component, blue: second component, red: third component). Second column: profiles as sampled in the 57 training subjects before edge-based alignment, but after initial registration. Third column: MAP estimate of component means and aligned profiles. The effect of alignment is especially clear when comparing panel 2 and 3 on the FA row; note the overall shift of about 1 mm. Columns 4–6: MAP estimates of mean and standard deviation for all components. Rows correspond to the modalities that were used in the model. Row 1: *T*_2_-weighted, row 2: *T*_1_-weighted, row 3: FA. Note that the observed profiles are shorter than the mean profiles; this corresponds to the lengths *k*′ and *k* in the main text.

An important question is whether the profile model, that is fitted for each vertex separately, is successful at finding a mean profile that captures the local intensity features and that is properly aligned with the physical boundary of the structure. Fig. 2 shows the unaligned profiles that were sampled for the different training subjects at a single vertex on the inferior boundary of the putamen. It also displays the prior and posterior means and the training profiles after they have been aligned to the posterior mean (i.e. at their most probable displacement *δ*_*si*_). It shows that, while the unaligned profiles are not closely aligned with each other, the aligned ones overlap much better; this is particularly clear for the FA profiles. In addition, the step in intensity is now located at the centre of the reference profile, which means that it is aligned with the prior that defines the physical boundary.

### Segmentation results

**Fig. 3:**
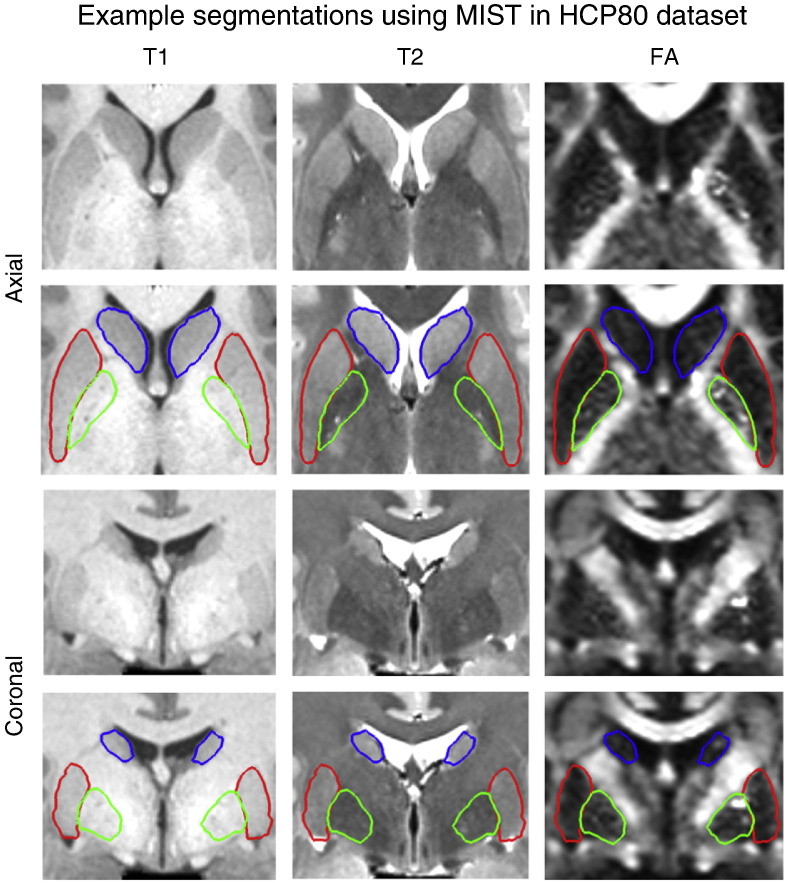
Example subject (499566) in the HCP80 dataset showing segmentations of putamen (red), globus pallidus (green) and caudate + nucleus accumbens (blue) on axial (top two rows) and coronal (bottom two rows) slices. The FA volume was not used for segmenting the caudate nucleus and nucleus accumbens.

The full set of segmented structures in an example subject is shown in Fig. 3. In this figure there are some clear examples of areas where multiple contrasts can complement each other. One example is the globus pallidus, which is essentially invisible on the *T*_1_-weighted image. Another example is the lateral boundary of the putamen, which, although there is contrast in the *T*_1_- and *T*_2_-weighted images, is difficult to see or detect due to the resolution of the volume. In the FA image, however, the boundary is much clearer. Further examples of segmentations in different subjects in the HCP80 dataset can be found in Figs. S1 (putamen), S2 (caudate nucleus and nucleus accumbens) and S3 (globus pallidus) in the Supplementary material. A comparison with the meshes produced by FIRST is given in Fig. S4.

**Fig. 4:**
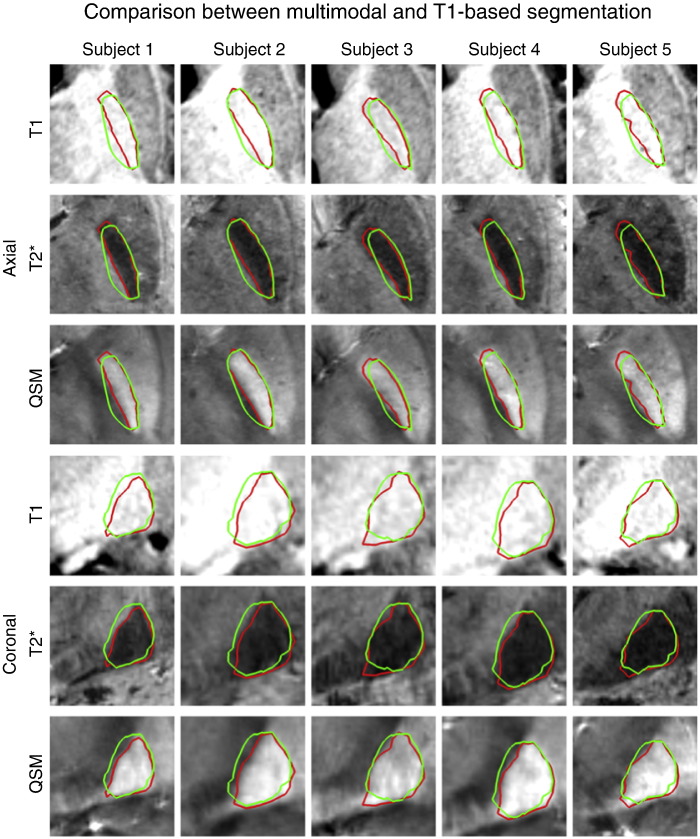
Segmentation results for the globus pallidus in the 7 T dataset using all three modalities (red) and *T*_1_-weighted only (green). Top three rows: axial slices, bottom three rows: coronal slices. The QSM volume was not used for segmenting the caudate nucleus and nucleus accumbens.

The advantage of multimodal segmentation for segmenting the globus pallidus can also be seen in the 7 T dataset. Fig. 4 shows the results obtained using the full multimodal model, as described in Table A.2, and a model using only the *T*_1_-weighted volume. The lateral boundary, which is clearly visible on the MP2RAGE scan, is accurately segmented in both cases, whereas the other boundaries improve with the inclusion of the other modalities. Examples of all three structures that are segmented are given in Fig. S5 in the Supplementary material.

### Voxel-based overlap with other methods

**Fig. 5:**
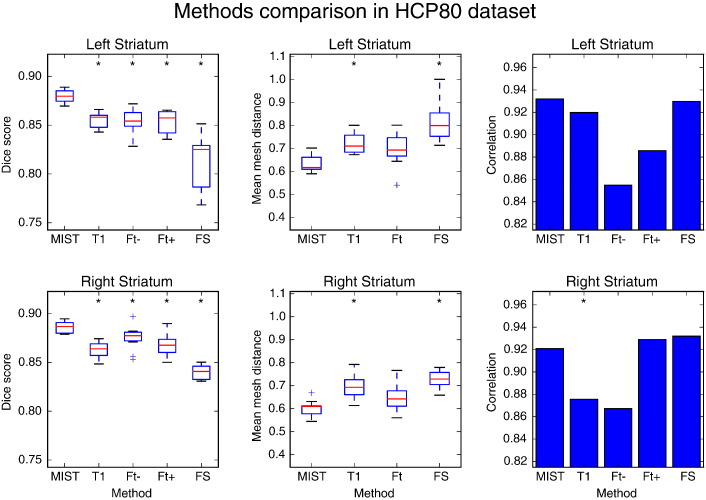
Dice overlap (first column) with manual segmentations and mean mesh distance in mm (second column) of segmentations produced by different methods in HCP 80 dataset (10 subjects). Correlation coefficients between manual and automatic mask volumes are shown in the third column. MIST: multimodal segmentation, T1: MIST with *T*_1_-weighted images only, Ft −/+: FIRST without and with boundary correction, FS: FreeSurfer. Data points that are outside the box by more than 1.5 times the interquartile range are treated as outliers. A significant difference in performance between a method and MIST is denoted by an asterisk (*p* ≤ 0.05, Wilcoxon signed rank test for the boxplots and Williams's test for the correlation coefficients. Computed using R, http://www.r-project.org/).

To quantitatively assess the performance of MIST, the results were compared with manually labelled scans. The Dice overlap scores for segmentation of the striatum in the HCP80 dataset using MIST, FIRST and FreeSurfer are shown in Fig. 5. The scores show that our method produces accurate segmentations in the HCP80 dataset with overlaps that are larger than those obtained with FIRST and FreeSurfer. Multimodal segmentation using MIST produces significantly more accurate results than segmentation using only the *T*_1_-weighted volume. The picture for the mesh-based distances is fairly similar, although the difference between MIST and FIRST is not significant in this case. Mesh distance is more sensitive than the Dice score to differences between segmentation in areas such as the tail of the caudate, a thin and elongated part of the structure.

In the comparison of volume correlations it is remarkable that boundary-corrected FIRST and FreeSurfer fare better than in the overlap- and distance-based comparisons. The advantage of comparing methods in terms of these correlations is that they show whether a method produces larger segmentations in subjects where a structure is larger, without being sensitive to the exact handling of the boundaries. In this case, the correlations indicate that FIRST and FreeSurfer capture the variability in striatal volume between subjects, despite the imperfections in boundary placement suggested by the Dice scores.

**Fig. 6:**
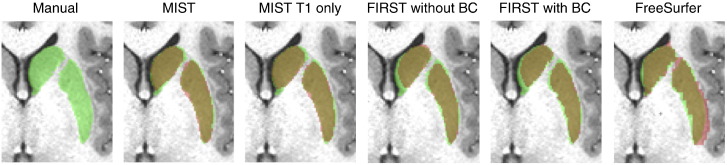
Example masks for the striatum using different methods in an example subject (499566) from the HCP80 dataset. Green: manual labelling, red: automatic segmentation, yellow: overlap. BC denotes boundary correction.

Examples of the voxel-based masks (as used to calculate the Dice scores) generated by the different methods are shown in Fig. 6.

**Fig. 7:**
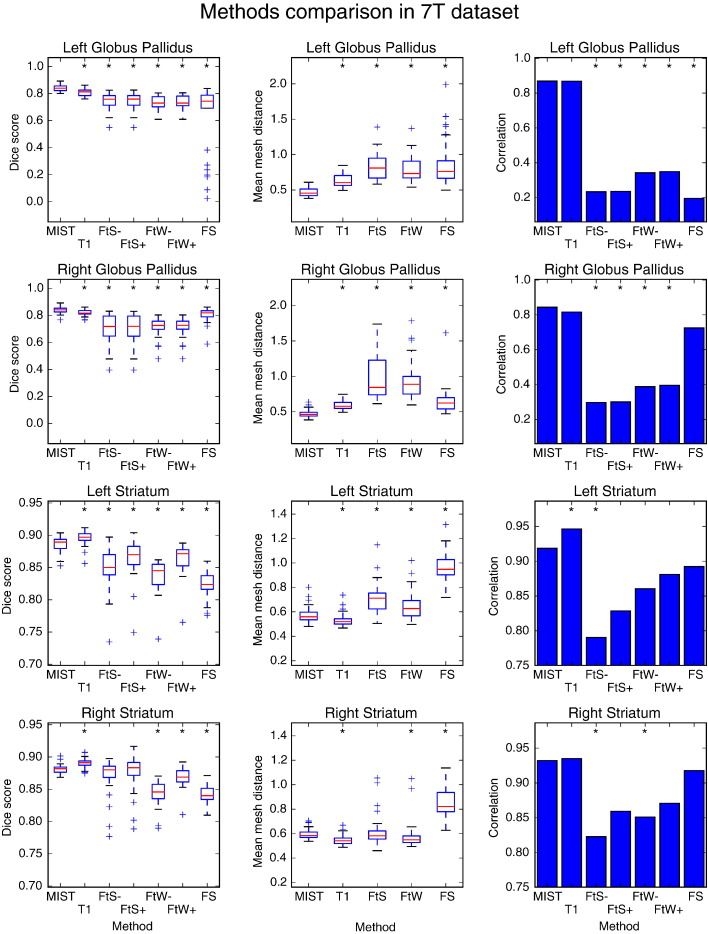
Dice overlap (first column) with manual segmentations and mean mesh distance in mm (second column) of segmentations produced by different methods in the 7 T dataset (29 subjects). Correlation coefficients between manual and automatic mask volumes are shown in the third column. MIST: multimodal segmentation, T1: MIST with *T*_1_-weighted images only, FtS −/+: FIRST without and with boundary correction on limited FOV MP2RAGE data, FtW −/+: FIRST without and with boundary correction on whole brain MP2RAGE data. Data points that are outside the box by more than 1.5 times the interquartile range are treated as outliers. A significant difference in performance between a method and MIST is denoted by an asterisk (*p* ≤ 0.05).

The overlap scores for the 7 T dataset are shown in Fig. 7. MIST again compares favourably to the other methods when segmenting the striatum, for both the multi-modal and *T*_1_-only cases. A striking feature of the results is that segmentation accuracy is also more consistent, in the sense that the difference between the lowest and highest scores is smaller. In the case of the globus pallidus, the improvement over the existing methods is even clearer than for the striatum. The correlation plots indicate that while FIRST and FreeSurfer show reasonable overlap with the manual segmentations, they do not accurately capture the differences in volume between subjects of the globus pallidus (in the left hemisphere only, in the case of FreeSurfer). For the globus pallidus, multimodal segmentation using MIST produces more accurate segmentations than segmentation using only the *T*_1_-weighted volume, whereas in the case of the striatum, which has clear contrast on a *T*_1_-weighted scan, MIST performs slightly better in the *T*_1_-only case.

### Application to HD dataset

**Fig. 8:**
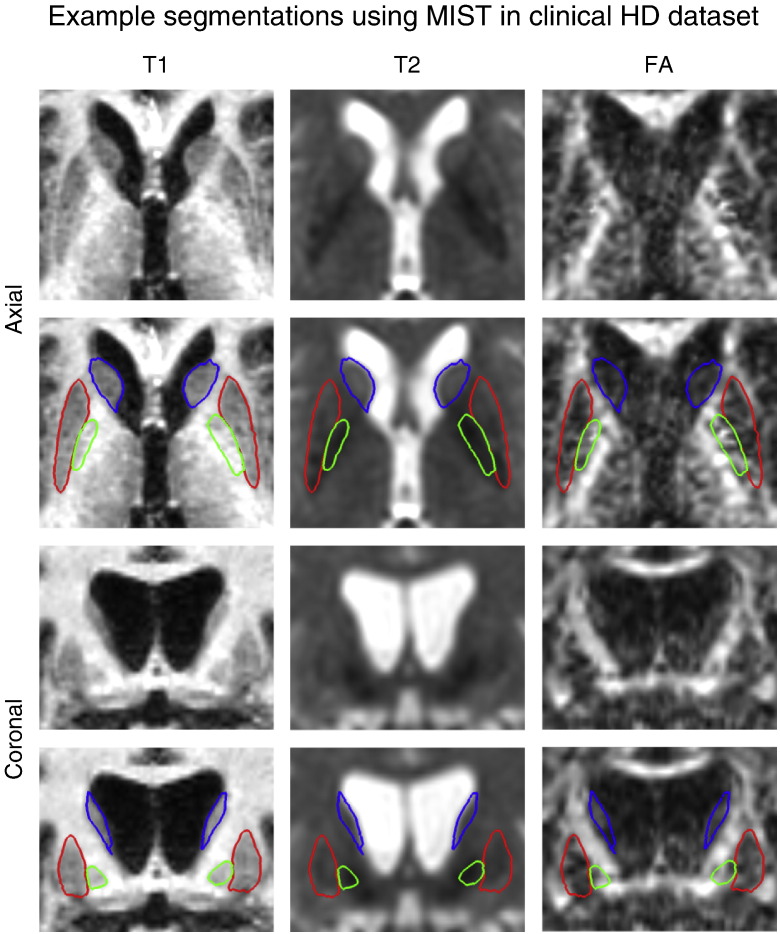
Example patient in the HD dataset showing segmentations of putamen (red), globus pallidus (green) and caudate + nucleus accumbens (blue) on axial (top row) and coronal (bottom row) slices.

Example segmentations in a patient in the HD dataset are shown in Fig. 8. This patient has significant atrophy of the subcortical GM. Despite this, MIST is successful in segmenting the different structures.

**Fig. 9:**
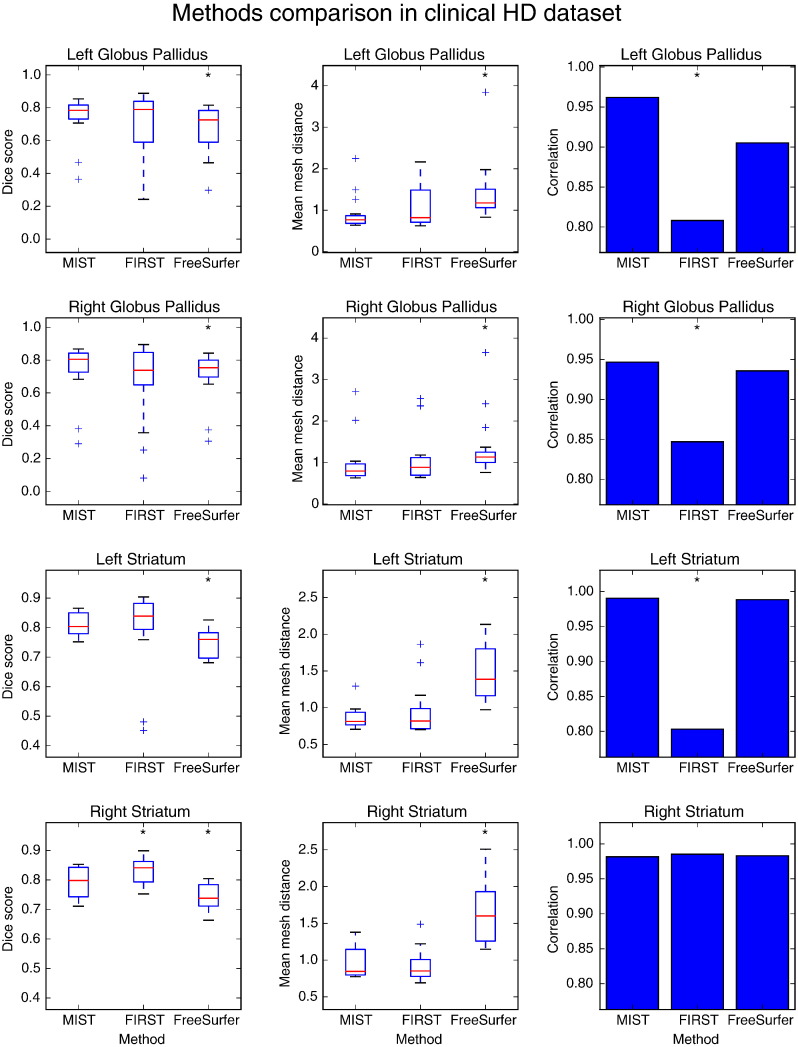
Dice overlap (first column) with manual segmentations and mean mesh distance in mm (second column) of segmentations produced by different methods in the HD dataset (16 subjects). Correlation coefficients between manual and automatic mask volumes are shown in the third column. Data points that are outside the box by more than 1.5 times the interquartile range are treated as outliers. A significant difference in performance between a method and MIST is denoted by an asterisk (*p* ≤ 0.05).

Fig. 9 shows the metrics of the segmentation performance of the different methods in the HD dataset. For the globus pallidus, MIST and FIRST perform comparably in terms of overlap and mesh distances and MIST performs slightly better than FreeSurfer (though statistically significant). In the comparison of volume correlations, MIST and FreeSurfer are comparable and FIRST shows significantly lower correlations.

For the striatum, Dice scores for MIST and FIRST are again higher (and mesh distances lower) than for FreeSurfer. The volumes of the FIRST segmentations of the left striatum correlate more weakly with the manual labellings than the volumes obtained with the other methods. This appears to be caused by two subjects in which FIRST performed poorly when segmenting this structure (see the Dice scores). This did not occur for the right striatum and this may explain the higher correlation in this case.

### General observations in the comparison between methods

The three measures of performance (Dice overlap, mean mesh distance and volume correlation) are sensitive to different aspects of the segmentations produced by the automatic methods, as illustrated above. MIST performs well in terms of all three measures in all datasets, while both FIRST and FreeSurfer are less consistent in this respect.

The methods comparison shows that FIRST achieves reasonable Dice scores for the globus pallidus in the 7 T dataset (above 0.7, Fig. 7), although the low volume correlations indicate that it is not successful in capturing between-subject variability. A possible explanation is that in the absence of image contrast, the segmentations are almost exclusively driven by prior knowledge. In this situation, there could still be substantial overlap of the segmentations with the globus pallidus due to the similarity of its appearance between subjects, but the variations themselves would not be captured by the segmentations. This would result in the low volume correlations. FIRST performs significantly better in the HD dataset, where contrast for the globus pallidus is much better in the *T*_1_-weighted volume.

The degree of asymmetry between left and right hemisphere results is remarkable. Volume correlations for the right striatum in the HCP80 dataset are significantly lower when using MIST with just the *T*_1_-weighted data compared to the multimodal case, while they appear similar for the left striatum (Fig. 5). As the testing dataset is relatively small (10 subjects), it is difficult to know whether this represents a real asymmetry in performance. A similar situation exists for the difference in the striatal volume correlation for FIRST in the HD dataset, which is significantly lower than for MIST in the left hemisphere only (Fig. 9). FIRST seems to perform poorly in terms of Dice scores in two subjects in the left striatum and it is hard to tell if the fact that no such outliers occur in the right hemisphere is caused by an actual asymmetry in the segmentation process or simply due to chance. A more convincing example of asymmetry is the performance of FreeSurfer in the globus pallidus in the 7 T dataset, which is substantially better in the right hemisphere (Fig. 7). We do not see an obvious explanation for this asymmetry in FreeSurfer's performance.

### Importance of the size of the training set

**Fig. 10:**
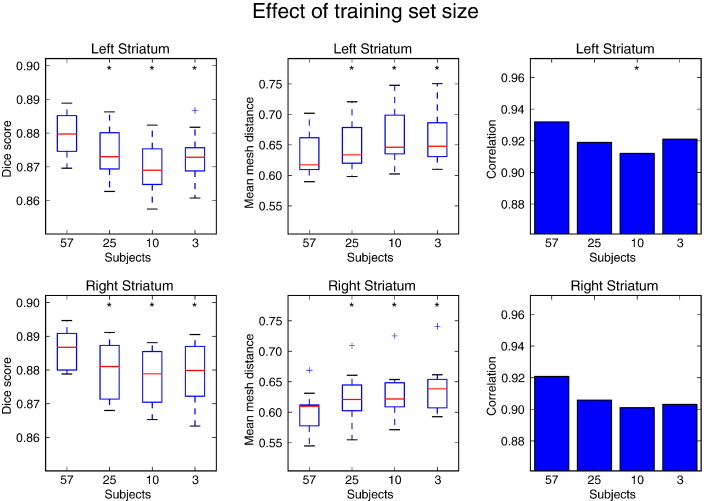
Dice overlap (first column) with manual segmentations and mean mesh distance in mm (second column) of segmentations produced after training on subsets of different sizes in the HCP80 dataset. Correlation coefficients between manual and automatic mask volumes are shown in the third column. Subsets of the full training set of 57 subjects were used to investigate how segmentation performance changes with smaller numbers of training subjects. A significant difference in performance between a subset and the full training set is denoted by an asterisk (*p* ≤ 0.05).

**Fig. 11:**
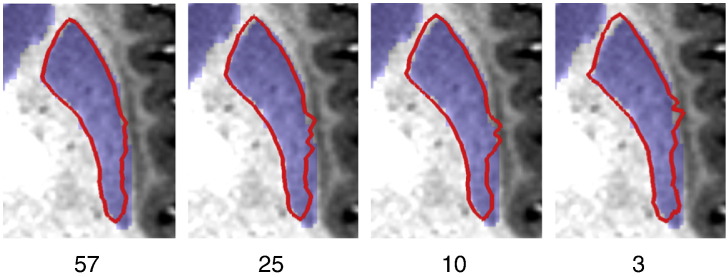
Segmentation of the putamen in an example subject in the HCP80 dataset for different numbers of training subjects. Red outline: automatic segmentation, blue overlay: manual labelling.

To investigate how sensitive segmentation performance is to the size of the training set, the striatum models for the HCP80 data were trained using a number of reduced-size subsets of the full training set. The results from this comparison are shown in Fig. 10. As expected, segmentations become less accurate with smaller training datasets, although performance in terms of these summary measures is still very reasonable even with the smallest training set. The reason for this behaviour is illustrated by Fig. 11. In this figure it can be seen that segmentation is successful for large parts of the structure even with very little training data by mostly relying on the intensity priors. In regions where anatomy is more complex, though, such as near the external capsule, learning the intensity profiles from the training data has a clear benefit. Another area where segmentation improves with more training area is the anterior end of the putamen. This may be caused by the islands of grey matter that are present between the putamen and caudate nucleus and whose appearance differs significantly between subjects and scans.

### Contribution of non-linear registration

**Fig. 12:**
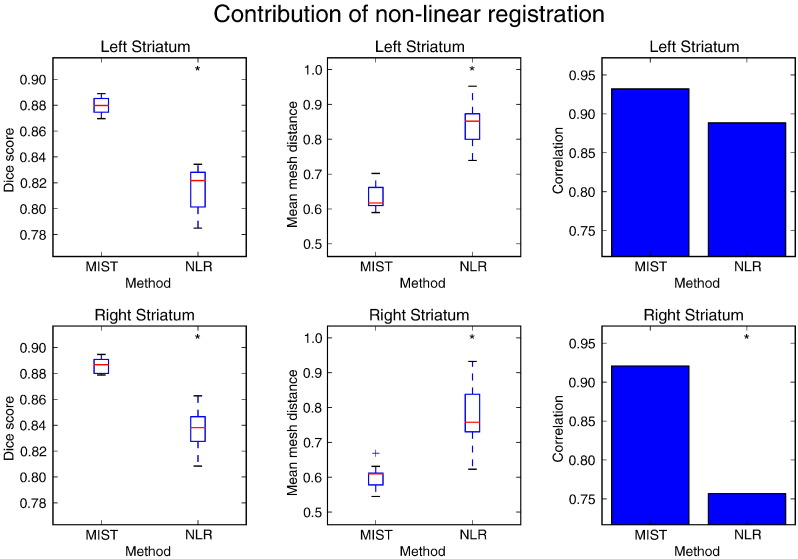
Dice overlap (first column) with manual segmentations and mean mesh distance in mm (second column) of segmentations produced in the HCP80 dataset using MIST and by using non-linear registration of the reference shape only (NLR). Correlation coefficients between manual and automatic mask volumes are shown in the third column. A significant difference in performance between NLR and MIST is denoted by an asterisk (*p* ≤ 0.05).

To investigate how large the relative contributions of non-linear registration and the subsequent segmentation steps are, Fig. 12 compares the result of the full segmentation procedure in the HCP80 dataset to the results obtained by applying non-linear registration to the reference shape without performing segmentation, i.e. setting all the displacements to zero. Non-linear registration produces a reasonable approximation to the structure, but the full segmentation procedure is required to obtain a high quality delineation.

## Discussion

### Segmentation results

The results in the previous section show that MIST can produce high-quality segmentations of the striatum and globus pallidus. The successful segmentation of the globus pallidus, which has poor contrast in a *T*_1_-weighted scan, illustrates the advantage of multi-modal segmentation. Although both the *T*_2_-weighted and FA contrasts provide more information on the boundaries of the structure, neither of these shows all aspects. The combination of the three images, however, allows the method to successfully segment the entire structure. This puts it at an advantage over FIRST and FreeSurfer, which only use a *T*_1_-weighted scan, and there is indeed an improvement in segmentation accuracy of the globus pallidus over these methods. MIST also produced good results in the 1.5 T HD dataset, confirming that the method works well with more typical clinical acquisitions as well. Contrast in the *T*_1_-weighted acquisitions in this dataset has been optimised for deep grey matter and as a result the globus pallidus is visible even on the *T*_1_-weighted images. This means conditions were probably fairly optimal for unimodal segmentation. MIST performs best of the three methods in terms of correlation with the manual segmentation mask volumes and comparably to the unimodal methods in terms of Dice overlap and mesh distances. This confirms that the method can handle more significant differences in anatomy than those that are typically present in healthy subjects.

Of the segmentations produced by the different methods that were evaluated, those produced by MIST showed the strongest volume correlations with the manual segmentations. This is an important result, as these correlations are probably more descriptive of the relevant properties of a method than the overlap and distance measures. Overlap and distance are very sensitive to issues of subjectivity, such as the preference of either a person doing manual segmentation or an automated method to include more or fewer boundary voxels. While such differences are important in quantifying the exact volume of a structure, they are not very relevant at all when the volumes are compared between groups and the question is just whether the volumes differ. A second scenario where correlation is the more relevant measure is when the numbers are to be used as predictor for some other measure, for example when using the volumes as a regressor in a general linear model.

MIST produced more consistent results than the other methods, especially in the HCP80 and 7 T datasets. It is difficult to make a definite statement about the cause of the difference, as the methodology underlying the three methods differs substantially. The larger number of images available to the multi-modal method is a clear advantage and this should make it more resilient to image noise. MIST was trained on images from the same population and with the same acquisition protocols as the images to be segmented. This is also likely to be advantageous, as this means that the trained model is more appropriate for the images that are segmented. Finally, considering that the largest improvements in segmentation quality and consistency were obtained in the HCP80 and 7 T dataset, it seems likely that the fact that the new method can more easily take advantage of the high quality data also contributes to better consistency.

MIST also performed well when used on only a *T*_1_-weighted image, in particular in the 7 T dataset. For the striatum, which is clearly visible on a *T*_1_-weighted scan, this may not be completely unexpected. Nevertheless, it confirms that the intensity model can reliably identify edges in the training data and that it is at least as sensitive to image information as the methods to which we compared. Fig. 4 shows that for the globus pallidus, the additional contrast is advantageous in producing good-quality segmentations. In Fig. 7, MIST performs slightly better on segmentation of the striatum when using only the *T*_1_-weighted data. The differences in the masks produced by multimodal and *T*_1_-only segmentation are rather subtle and it is difficult to conclusively determine what causes this difference. The effect of partial volume averaging and data smoothness will be slightly different in the multimodal case and due to the fact that there is more intensity data, the relative weighting of the shape prior will be somewhat lower. It is likely that the difference is caused by a combination of these effects. Both multimodal and *T*_1_-only MIST show better consistency than the other methods. It should also be kept in mind that the manual segmentations were done on the *T*_1_-weighted data, meaning that they have not taken the additional information in other modalities into account.

The DWI protocol used in the HCP dataset has a total acquisition time of about an hour, but it is useful to note that for purposes of subcortical segmentation, high angular resolution and multiple *b*-values are not required and acquisition could be much quicker, as long as the spatial resolution is relatively high. It should also be noted that while the acquisition protocol for the *T*_1_-weighted scan in the HCP data has been optimised heavily for cortical contrast, subcortical contrast is significantly poorer.

### Parameter setup

Using the script that is described in Appendix A, MIST can be retrained fully automatically on any dataset that contains one or more of the contrasts that were used in this paper or similar contrasts. If the dataset contains fundamentally different contrasts, the user can add priors for these modalities to the generated setup. The fact that the method can be easily retrained eliminates bias that might otherwise arise when the model is applied to a population that is different from the one it was trained on. This could be an important advantage in patient studies, where brain anatomy could be substantially different from the healthy population. If desired, a completely unbiased result could be obtained by creating a reference mesh from the study average image; this would require a manual labelling of only that image. Note that, while in this paper we used separate training and evaluation groups, in applications there would be no problem with training on a balanced and representative subgroup of the study subjects.

A suitable specification of the priors is essential in order to train the model to achieve good quality segmentations. This specification should reflect our a priori belief about the shape and amplitude of an intensity profile, which is determined by two factors: Anatomical variability and image properties. The first of these two, the anatomy and the anatomical differences between subjects, are what dictates the choice of function to be used as a prior (see Table 1). In the case where we expect only a single image edge to be present in the measured profiles, specifying the prior profile would be simple, as a simple step function (i.e. *f*_*step*_ in Table 1) with the intensity inside the structure as the first parameter and the outside intensity as the second parameter represents this situation accurately. In reality, however, there are likely to be additional image features that we are not interested in, such as edges farther away from the boundary of the structure of interest. The advantage of the exponential shape, which we have mostly used, is that it contains one sharp edge, which helps alignment, but not any other edges that might cause the method to unintentionally align to other structures. The advantage over the step function, which also contains only one edge, is that the amplitude returns to the value for the inside (left) section of the profile. This is beneficial in assigning profiles to a mixture component, as it reduces the influence of intensity difference that are not related to the edge feature.

The second factor in specifying the priors is finding an initial estimate of the image intensities. These intensities can vary significantly between acquisitions and scanners and we have used the automated setup script described in Appendix A to find these for the different datasets presented in this paper. The script uses rules based on the intensities inside different structures to find suitable values to be used in the prior specifications. This should automate the retraining process for the striatum and globus pallidus in datasets that have the types of contrasts as used here. Fundamentally different contrasts (i.e. other than *T*_1_-, *T*_2_-weighted, FA or QSM) that show different aspects of structures will require the user to manually specify the shapes and intensities of the prior profiles for those contrasts. To segment a completely new structure (not the striatum or globus pallidus), the user will need to provide a reference mesh and specify the profile priors manually (or extend the Python setup script).

### General considerations

By considering displacements along the shape normals only, we were able to formulate a model that is relatively straightforward and in which the intensity models factorise if the shape model is not taken into account. As mentioned in the introduction, this type of deformation was chosen because most of the intensity information that is relevant to find the location of the structural boundary is along the normals. The results we obtained suggest that with an appropriately constructed reference shape, this parameterisation is flexible enough to capture the anatomical variability that is present.

The meshes that we have used are relatively regular in terms of point spacing and triangle areas. We have chosen to do this, as we want the model to be able to capture small and unique variations like vessels with the same resolution anywhere along the surface. It is conceivable that in areas of high curvature a higher mesh resolution would be advantageous, but for the structures that we segment in this paper we did not see any resolution-related problems with the current meshes. Using lower resolutions for parts of the mesh would compromise our aim of being able to capture small variations anywhere along the mesh and instead impose a degree of regularity.

The primary purpose of the shape model is to regularise segmentations, as the displacements that are determined for all vertices independently can be noisy in areas of weaker contrast. To overcome this problem, the shape model learns the mean displacement at each vertex and the covariance between vertices. This regularises the segmentations by only generating large displacements with respect to the mean learned from the training data if the images provide strong enough evidence in terms of edge contrast. It also favours segmentations that respect the covariance between displacements observed in the training data and the smoothness specified by the covariance prior.

For further statistical analysis, there are two forms of output that can be used. The first option is to use the vertex-wise displacements. These are generated in MNI coordinates, but as the nonlinear transformation from native to MNI coordinates already includes some of the shape variability, the best option appears to be to undo the nonlinear part of the transformation, but not the affine part. This means that global scaling factors, which primarily represent head size, are not included in the displacements, but that all local deformations are included. Standard multivariate analysis tools can then be used for inference. A second option is to use the voxel-based output for further analysis. This can be a good approach to get only a scalar volume for each structure, or to get a mask that can be used to define a region of interest (ROI) for further analysis.

We chose to treat the caudate nucleus and the nucleus accumbens as a single structure. There is virtually no contrast between these structures in the images that were used here. Because of this, a subdivision would not be driven by the data, but rather it would be based solely on some form of prior knowledge. In the case of a shape-based analysis using the vertex-wise displacements, this may not be a problem, as different patterns of variation within the merged structure can still be represented. In the case of a voxel-based further analysis, we propose to use a subdivision based on the MNI coordinates if this is required.

The runtime of the training stage of the method depends on many factors, the most important of which are the size of the training dataset and the number of modalities. Evaluation time of the cost function scales linearly in both factors, although total runtime might deviate from this linear behaviour due to the non-linear optimisation. Training the method on the structures and datasets presented in this paper took between an hour and half a day of processor time on recent PC hardware. In practice, we have parallelised the training stage, which means the wall time spent is much lower. Segmenting a new image after training takes under a minute on a single processor.

In this paper, we have described a flexible new method for subcortical segmentation. It can successfully combine information from multiple modalities and can be retrained for datasets with different characteristics. We have applied the method to the striatum and globus pallidus, but the framework is more general and can be applied to other structures. Retraining is automatic for the structures and contrast types considered in this paper. The method can be applied to new structures and contrasts by the user through the specification of suitable priors and a reference mesh in the case of new structures. The implementation of the method that was used to produce the results in this paper will be made publicly available through inclusion in a future release of FSL (http://fsl.fmrib.ox.ac.uk) and will be called MIST.

## Figures and Tables

**Table A.2:** Intensity model parameter template. Each row specifies one or more priors. The inside intensity corresponds to the left half of the prior profile in Fig. 2 and the outside intensity corresponds to the peak value of the prior profile. ‘Self’ denotes the median intensity inside the structure to be segmented. Where an amplitude multiplication factor is given, the size of the peak is scaled by that factor.

Structure	Modality	Type	Inside	Outside
Putamen	*T*_1_	Exponential (1 and 4 mm)	Self	Average of GP and WM
Exponential (1 mm)	Self	Self × 0.5
*T*_2_	Exponential (1 and 4 mm)	Self	Average of GP and WM
Exponential (1 mm)	Self	Self × 1.5
FA	Exponential (4 mm)	0.1	0.7
Exponential (1 mm)	0.1	0.4
Flat	0.1	
QSM	Exponential (2 mm)	0.1	0.2
Step	0.1	0.0
Flat	0.0	
Globus pallidus	*T*_1_	Exponential (3 mm)	Self	Putamen
Exponential (3 mm)	Self	WM (amplitude × 3)
*T*_2_	Exponential (3 mm)	Self	Putamen
Exponential (3 mm)	Self	WM
FA	Step	0.2	0.6
Flat	0.2	
QSM	Step	0.2	0.1
Step	0.2	0.0
Caudate and accumbens	*T*_1_	Step and exp. (1 and 4 mm)	Self	Ventricles (amplitude × 1.5)
Step and exp. (1 mm)	Self	WM (amplitude × 0.67)
*T*_2_	Step and exp. (1 and 4 mm)	Self	Ventricles
Step and exp. (1 mm)	Self	WM

**Table A.3:** Prior specifications of standard deviation; *β* is the scalar value that is used for all coefficients of the covariance priors and *f* is the relative resolution of the modality. See the main text for a description of how these values are used in specifying the model.

Modality	Prior standard deviation ($\sqrt{\beta /f}$)
*T*_1_	Self × 0.1
*T*_2_	Self × 0.1
FA	0.1
QSM	0.02

**Table A.4:** Shape and intensity model parameters. The Wishart shape parameters can be thought of as the number of pseudo-observations that the prior covariance represents. The parameter *σ*_*δ*_ is only used when the intensity models are trained in isolation.

Part of model	Parameter	Symbol	Value
Both	Step size	*ℓ*	Half of voxel size
Number of steps	Δ	2 × ⌊3mm/*ℓ*⌋
Shape model	Width parameter	*w*	0.6
Height parameter	*h*	0.05
Weight of prior mean	*n*_0_^*s*^	10
Wishart shape parameter	*α*^*s*^	$\frac{N-1}{2}+3$
Intensity model	Weight of prior mean	*n*_0_	10
Wishart shape parameter	*α*^0^	$\frac{k-1}{2}+3$
Dirichlet parameter for all components	*α*	2.0
Width of prior on *δ*	*σ*_*δ*_	5.0
Smoothness	*σ*^*I*^	0.5

**Table 1:** Functions used to specify mean and covariance priors; *x* is the distance along the profile, *x* = 0 at the physical boundary of the structure and *a*, *b* and *v*, *w* are the parameters as specified by the user. The exponential prior has a step in intensity at *x* = 0 and decays back to the original intensity for positive *x*.

Description	Function	
Flat	*f*_flat_(*a*) =	*a*
Step	*f*_step_(*a*, *b*) =	$\left\{\begin{array}{ll} a & \mathrm{if}\;x<0,\\ b & \mathrm{if}\;x\geq 0 \end{array}\right)$
Exponential	*f*_exp_(*v*, *a*, *b*) =	$\left\{\begin{array}{ll} a & \mathrm{if}\;x<0,\\ a+\left(b-a\right)e^{-\frac{x}{v}} & \mathrm{if}\;x\geq 0 \end{array}\right)$

## Appendix A Parameter setup

In order to make it as straightforward as possible for users of our method to retrain the models on new datasets, we use a script that automatically sets all the parameters by combining prior anatomical knowledge with properties of the training images. This script was also used to set the parameters for all datasets presented in this paper.

Two pieces of prior anatomical knowledge are required by the model: A reference mesh, the generation of which was described in the main text, and the specification of the profile priors. While the intensities of the priors depend strongly on the acquisition, their shapes (see Table 1) are determined by anatomical features like the number of different neighbouring structures and their sizes and shapes. It is therefore possible to define a set of reference meshes and rules for setting up the priors that should apply to any dataset and combine these with intensities that can automatically be determined based on the images. In this section we describe the template used by the automatic setup script to generate a full set of parameters that should apply to any dataset that contains similar modalities to the ones described here, or a subset of them.

The intensities are found automatically by the script from the user's dataset by extracting the intensities inside regions of interest (ROIs). The ROI masks are derived from the Harvard–Oxford subcortical atlas that is provided with FSL. The median of all values with above 75% probability in the atlas is extracted from each subject and the mean of these medians is the value filled in into the templated defined by Table A.2. It should be noted that, as the intensity setup is derived from the image, each component in the table can be used with a range of similar contrasts. In this paper, for example, the *T*_2_- and *T*_2_^⁎^-weighted images in the different datasets have quite different appearances, yet they can all use the *T*_2_ setup from the table. The same is true for the different *T*_1_-weighted scans.

The prescriptions for the profile priors that were used for all datasets in this paper are displayed in Table A.2. The shapes of the functions have been specified manually through considering the anatomy of the structures' boundaries and some experimentation.

For the *T*_1_ and *T*_2_ modalities in the putamen, priors are specified for two cases: The case where the neighbouring structure at a vertex is either the globus pallidus or WM and the case where there is a vessel instead. For the first case, two exponentially decaying priors are specified to better capture the range of profiles that may be observed. For example, on the lateral side of the putamen there is the external capsule, a thin sheet of WM, while the globus pallidus on the medial side is a much thicker structure. The diameter of vessels is typically small, which is why the prior that is specified to handle them decays quickly. We have used a single outside intensity for the globus pallidus and WM as the intensity changes with respect to the putamen are the same for both (i.e. higher on *T*_1_-weighted and lower on *T*_2_-weighted volumes).

For the FA and QSM modalities, wide and narrow priors are specified for the same reasons as in the *T*_1_ and *T*_2_ modalities. As vessels are not as clear on these modalities, no vessel prior is included for these modalities. The QSM prior with a positive edge is included for the boundary with the globus pallidus, whereas the negative edge should handle boundaries with WM. The intensities for the FA and QSM modalities are not derived from the images as the former has a fixed range and the latter is quantitative.^2^

The priors for the globus pallidus are set up to represent two cases: On the medial side, the structure borders the WM and on the lateral side the pallidum. Variations in thickness are less of an issue here, as are vessels, so the prior specification is simpler. The reason for including priors with two different inside intensities for QSM is that this is one of the few cases where the inside intensity varies quite significantly across the structure.

For the combined caudate and accumbens mesh, only the *T*_1_ and *T*_2_ modalities were used. The reason for this is that there is very little contrast in the FA and QSM images between the caudate nucleus and the ventricles, while there is strong contrast between the ventricles and the corpus callosum. This may result in ambiguities if the ventricles are small. The priors for the caudate nucleus and nucleus accumbens were set up in much the same way as for the putamen, but using the WM and ventricles as reference structures. As these structures can be very wide as seen from the caudate nucleus, step priors were added as well.

While the model allows the specification of more complex functions, in practice we will always use a flat profile with height *β* (see Eq. (13)) to specify the coefficients for the prior covariance. Up to a smoothness-dependent constant, the square root of *β* behaves like the standard deviation. We set up the prior value of this standard deviation as a fraction of the observed image intensity. Note that we cannot use the image signal-to-noise ratio, as the variability of the profile is a composition of image noise and anatomical variability. The value that is derived from the image is scaled by the factor in Table A.3 and by an additional factor *f*, which is the resolution of the modality divided by the highest-resolution modality. This resolution scaling factor is used to weight the modalities according to the smoothness, which is higher in low-resolution images; see the discussion about *σ*^*I*^ below.

The remaining parameters, including the shape model parameters, are set up according to Table A.4. We have used the same values for all datasets presented here and we believe it to be likely that there will be little need to change most of these when applying the method to other datasets. The most important parameter is the number of steps, which together with the step size determines the maximum displacement that can be fitted at each vertex. The setting in the table amounts to an even number of steps that corresponds to a maximum displacement of 3 mm inwards or 3 mm outwards. This appears to be sufficient in practice even in the HD dataset. The smoothness parameter *σ*^*I*^ can be used to avoid over-weighting lower resolution modalities, although in practice we do this using the variance priors described above. This has a similar effect but has the advantage that it does not interfere with non-linear optimisation by enhancing the collinearity between parameters.

The parameters *w* and *h* control the prior variance and covariance between vertices. They can be used to control the smoothness of the final segmentation. It is important to note however, that this smoothness concerns the displacements with respect to the mean shape and as we train all vertices in isolation, there is no guarantee that this mean will be smooth. The parameters are useful to regularise the model in the case of noisy data, however. The final segmentation result is not very sensitive to changes of *w* and *h* around their present values. Low values of *w* and high values of *h* reduce the regularisation effect, while low values of *h* constrain the mesh to the mean. Very high values of *w* may interfere with non-linear optimisation as this causes the parameters to be highly collinear. As the meshes used for the different structures in this paper have similar numbers of vertices per unit of surface, we expect similar smoothness and the same values for *w* and *h* are used for all of them.

Weighting of the priors relative to the training data is controlled by the parameters *n*_0_^*s*^, *α*^*s*^, *α*_0_ and *n*_0_. We have set these close to their minimum values as there is no obvious advantage to increasing these weights. One reason for setting higher values might be if stronger regularisation using *w* and *h* is desired. Finally, the parameter *α* determines whether the intensity model will prefer mixtures where one component explains all observations versus mixtures with more equal mixing. The current setting gives a slight preference to the first case, although in practice the effect of this parameter appears limited.

## Footnotes

1 Dice’s coefficient is defined as $\frac{2N_{\mathrm{AB}}}{N_{A}+N_{B}}$, where *N*_*A*_ and *N*_*B*_ are the number of voxels in masks *A* and *B*, and *N*_*AB*_ is the number of voxels in the overlapping region (Dice, 1945).
