# Supplementary material for: Automatic segmentation of the striatum and globus pallidus using MIST: Multimodal Image Segmentation Tool
Source: Neuroimage. 2016 Jan 15;125:479–97. doi: 10.1016/j.neuroimage.2015.10.013 (PMC4692519; doi:10.1016/j.neuroimage.2015.10.013)
Supplement: Supplementary file 1 — Supplementary material. [file mmc1.pdf]

Supplementary material for 'Automatic segmentation of the striatum and globus pallidus using MIST: multimodal image segmentation tool'

October 19, 2015

1 Segmentation examples in individual subjects

Example segmentation results for the left putamen in five subjects from the HCP80 dataset are shown in Fig. S1. The segmentation procedure used the T_1-weighted, T_2-weighted and FA images, but only the T_1-weighted volume is used for visualisation. Results for the combination of the caudate nucleus and nucleus accumbens are shown in Fig. S2. In this case, T_1- and T_2-weighted images were used for segmentation, as these contained adequate contrast. In addition, as the ventricles are invisible on an FA image, the corpus callosum represented the closest image edge for parts of the caudate nucleus; this introduced an ambiguity in finding the actual boundary of the caudate nucleus.

Results for the globus pallidus in the same dataset are displayed in Fig. S3. This is an example of a structure where there is no adequate contrast in the T_1-weighted volume. In the coronal slices, the superior boundary is clearly visible in the T_2-weighted volume. The medial boundary is not as clear on this volume and appears to be detected mainly on the basis of the FA-contrast.

Figure S4 shows a qualitative comparison of MIST with the mesh-based output from FIRST. MIST compares favourably and clearly improves on the output from FIRST in a number of areas. The segmentation of the globus pallidus illustrates the advantage of multimodal segmentation; FIRST fails to estimate the medial boundaries of the structure, whereas the added contrast in the T_2-weighted and FA images allow the new method to correctly estimate all boundaries.

Examples of the structures that were segmented in the 7T dataset are shown in Figure S5. The additional contrast offered by the high resolution T_2^*-weighted images, as well as the QSM ones, offers a clear advantage over segmentation based on just the T_1-weighted volume.

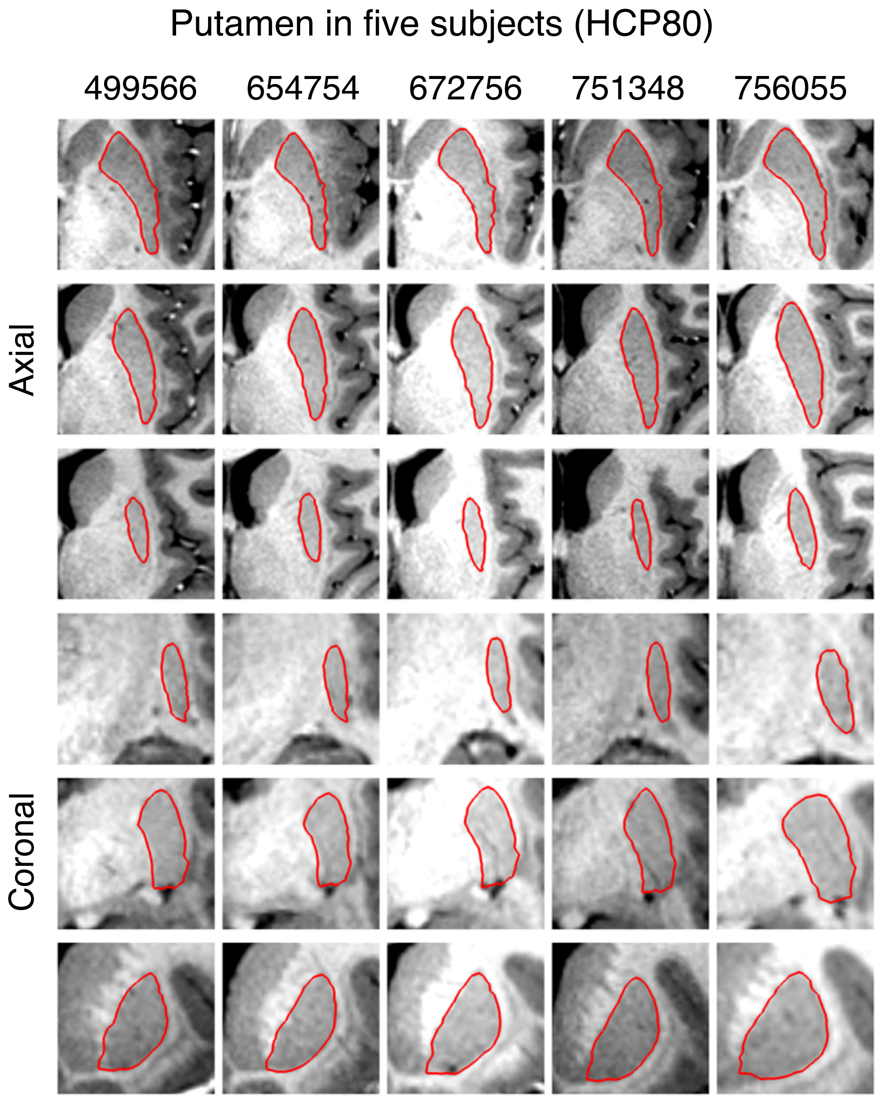

Figure S1: Segmentation of the left putamen in the HCP80 dataset in five subjects. Top three rows: axial slices, bottom three rows: coronal slices.

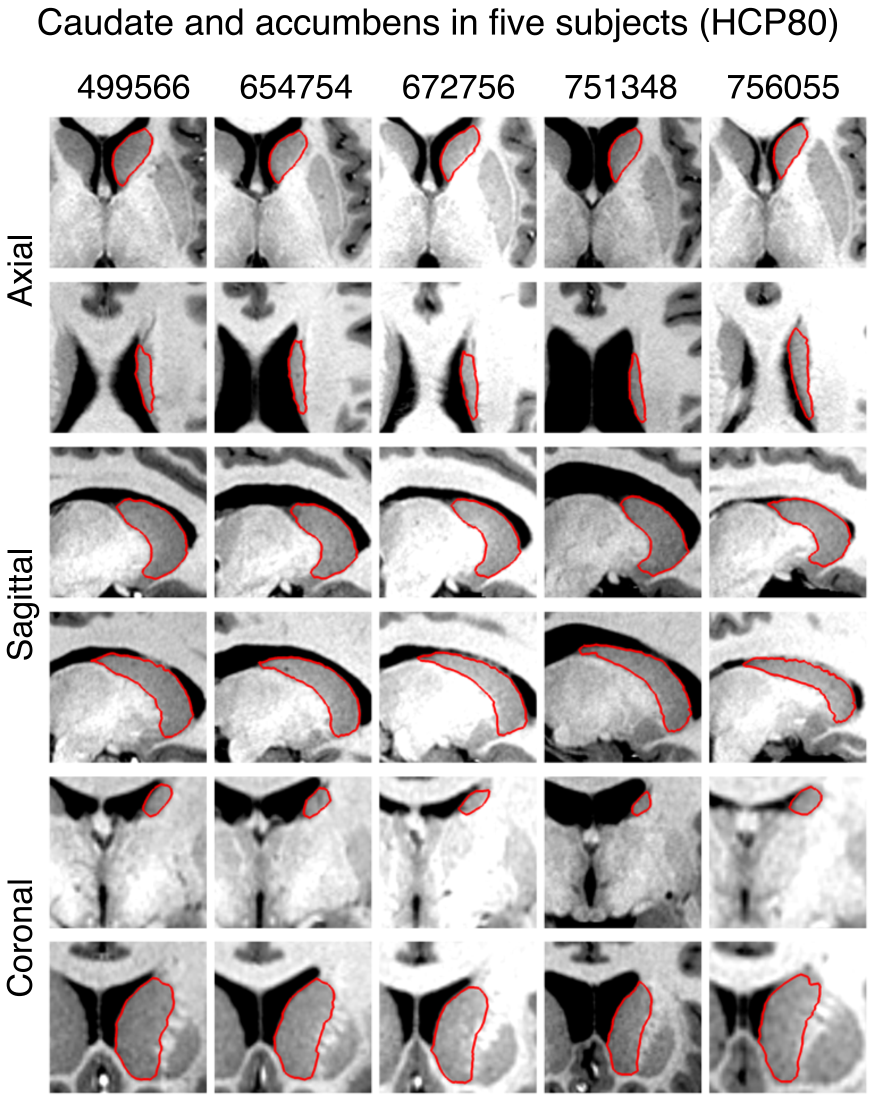

Figure S2: Segmentation of the left caudate nucleus and nucleus accumbens in the HCP80 dataset in five subjects. Top two rows: axial slices, middle two rows: sagittal slices, bottom two rows: coronal slices.

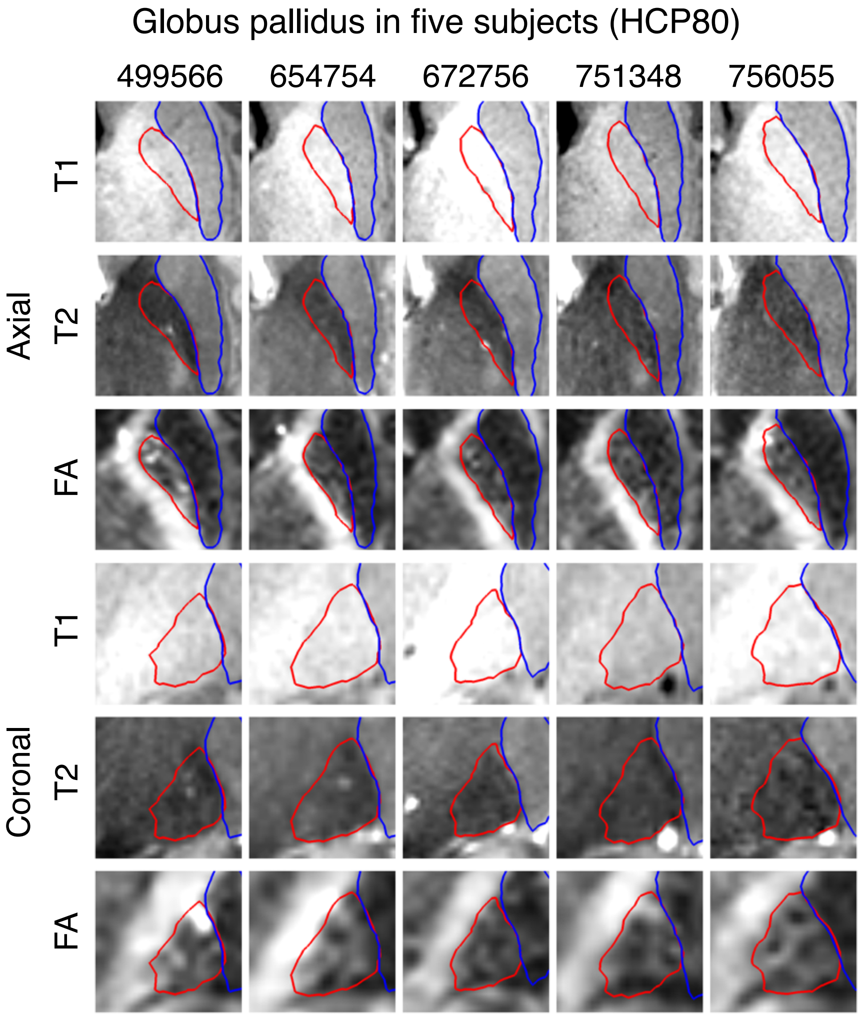

Figure S3: Segmentation of the left globus pallidus in the HCP80 dataset in five subjects, overlayed on the T_1-weighted, T_2-weighted and FA volumes. Top three rows: axial slices, bottom three rows: coronal slices. The putamen is shown in blue for reference.

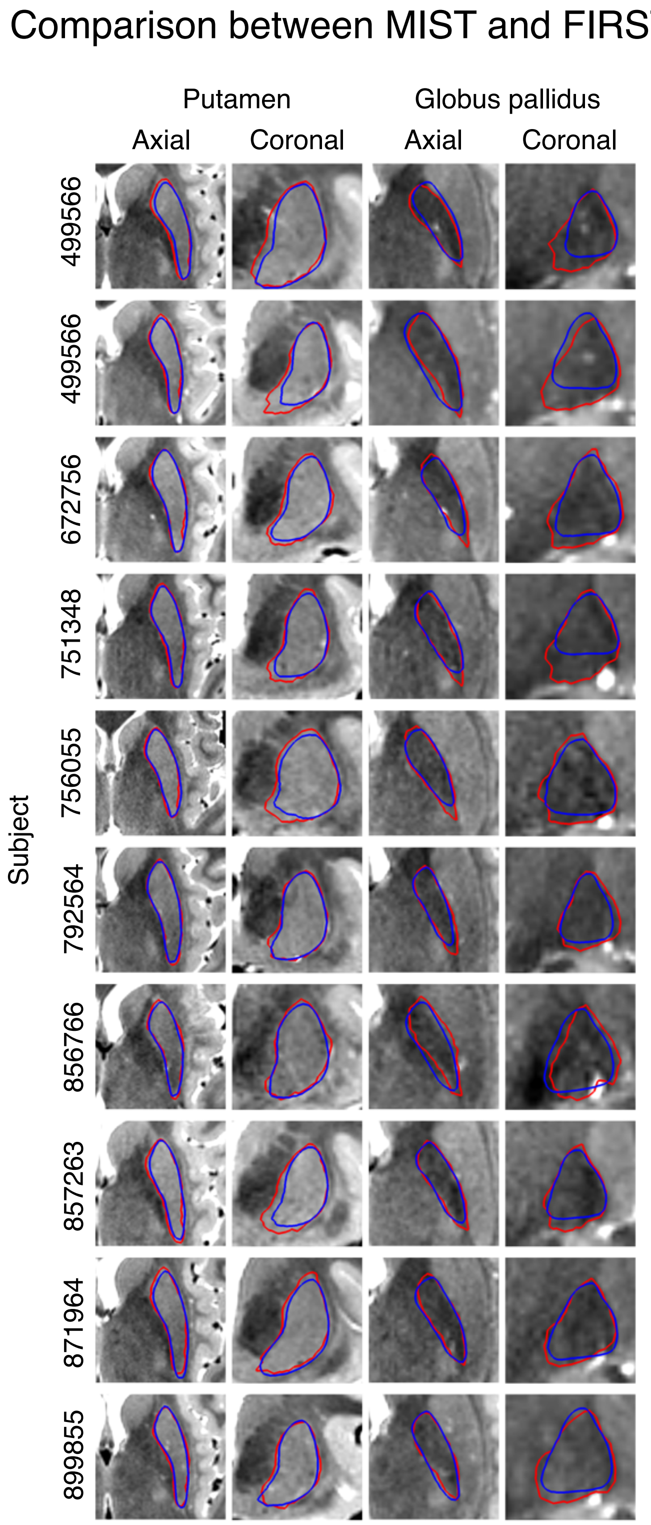

Figure S4: Comparison between MIST (red) and FIRST (blue) of segmentations of the putamen and globus pallidus in ten subjects of the HCP80 dataset, displayed on the T_2-weighted volume. Columns 1 and 3: axial slices, columns 2 and 4: coronal slices.

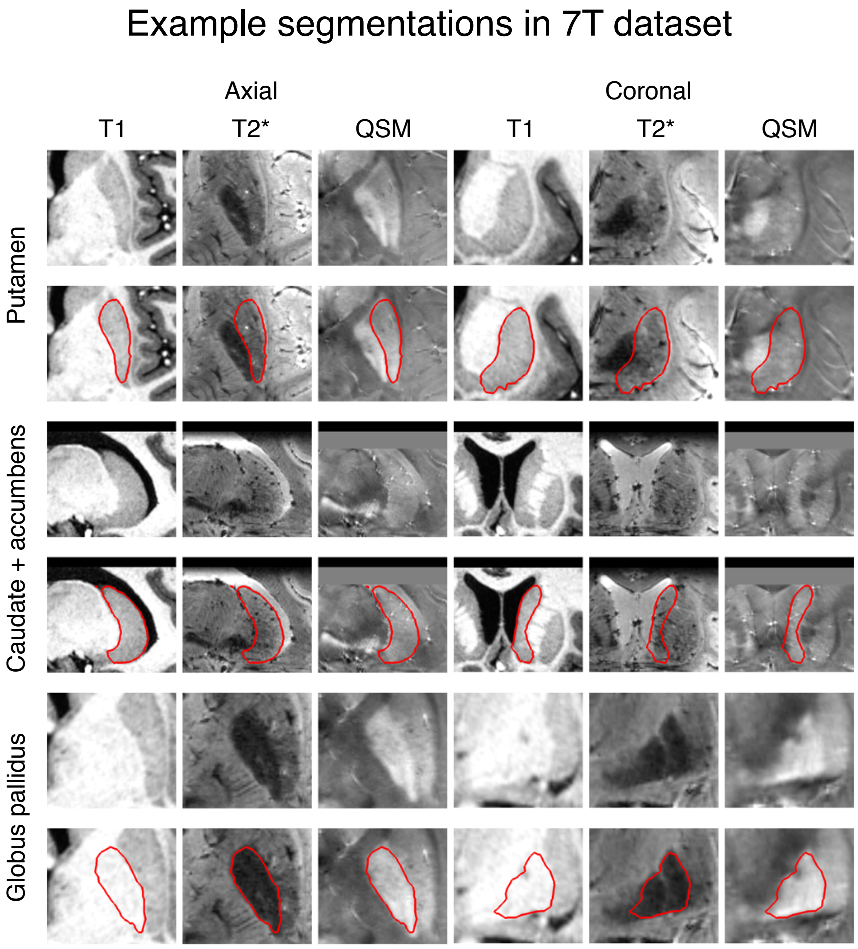

Figure S5: Segmentation of the putamen (left: axial, right: coronal), the caudate nucleus and nucleus accumbens (left: sagittal, right: coronal) and the globus pallidus (left: axial, right: coronal) in one subject in the 7T dataset, overlaid on the T_1-weighted, T_2^*-weighted and QSM volumes.

2 Details of the intensity model

Logarithm of the probability and its derivatives The logarithm of the probability Eq. 17 itself is

$$\log p(M, L, \theta, \delta_1, \dots, \delta_S | \mathbf{z}_1, \dots, \mathbf{z}_S)$$
$$= \text{const.} + \sum_{m=0}^{N_r} \sum_{r=0}^{N_r} \log \mathrm{N}(\mu_{mr} | \mu_{mr}^0, n_0 \mathbf{\Lambda}_{mr})$$
$$+ \log \mathrm{Wi}(\mathbf{\Lambda}_{mr} | \alpha^0, \beta_{mr}^0) + \sum_{m=0}^{N_m} \log \mathrm{Dir}(\theta_m | \alpha)$$
$$+ \sum_{s=0}^{N} \log \left(\sum_{\delta=0}^{\Delta} \mathrm{N}(\delta | \mu_\delta, \lambda_\delta) \prod_{m=0}^{N_m} \sum_{r=0}^{N_r} \mathrm{N}(\mathbf{z}_{sm} | \mu_{mr}^\delta, \mathbf{\Lambda}_{mr}^\delta) \mathrm{Cat}(r | \theta_m) \right). \quad (1)$$

The derivatives with respect to its parameters are

$$\frac{\mathrm{d}}{\mathrm{d}\theta_{nj}} \log p(M, L, \theta, \delta_1, \dots, \delta_S | \mathbf{z}_1, \dots, \mathbf{z}_S) =$$
$$\sum_{s=0}^{N} \frac{1}{Z_s} \left[\sum_{\delta=0}^{\Delta} \mathrm{N}(\delta | \mu_\delta, \lambda_\delta) \left(\prod_{\substack{m=0 \\ m \neq n}}^{N_m} \sum_{r=0}^{N_r} \mathrm{N}(\mathbf{z}_{sm} | \mu_{mr}^\delta, \mathbf{\Lambda}_{mr}^\delta) \mathrm{Cat}(r | \theta_m) \right) \right.$$
$$\left. \times \left(\mathrm{N}(\mathbf{z}_{sn} | \mu_{nj}^\delta, \mathbf{\Lambda}_{nj}^\delta) - \mathrm{N}(\mathbf{z}_{sn} | \mu_{nk+1}^\delta, \mathbf{\Lambda}_{nk+1}^\delta) \right) \right]$$
$$+ \frac{1 - \alpha_{k+1}}{1 - \sum_{\ell=1}^{k} \theta_{n\ell}} + \frac{\alpha_j - 1}{\theta_{nj}} \quad (2)$$

for the mixing coefficients,

$$\frac{\mathrm{d}}{\mathrm{d}\mu_{nj}} \log p(M, L, \theta, \delta_1, \dots, \delta_S | \mathbf{z}_1, \dots, \mathbf{z}_S) =$$
$$\sum_{s=0}^{N} \frac{1}{Z_s} \left[\sum_{\delta=0}^{\Delta} \mathrm{N}(\delta | \mu_\delta, \lambda_\delta) \left(\prod_{\substack{m=0 \\ m \neq n}}^{N_m} \sum_{r=0}^{N_r} \mathrm{N}(\mathbf{z}_{sm} | \mu_{mr}^\delta, \mathbf{\Lambda}_{mr}^\delta) \mathrm{Cat}(r | \theta_m) \right) \right.$$
$$\left. \times \mathrm{Cat}(j | \theta_n) \mathrm{N}(\mathbf{z}_{sn} | \mu_{nj}^\delta, \mathbf{\Lambda}_{nj}^\delta) \mathrm{U}_\delta \left((\mathbf{z}_{sn} - \mu_{nj}^\delta)^T \mathbf{\Lambda}_{nj}^\delta \right) \right]$$
$$- n_0 (\mu_{nj}^T - {\mu_{nj}^0}^T) \mathbf{\Lambda}_{nj} \quad (3)$$

for the component means and

$$\frac{\mathrm{d}}{\mathrm{d}\boldsymbol{\Lambda}_{nj}} \log p(M, L, \theta, \delta_1, \ldots, \delta_S | \mathbf{z}_1, \ldots, \mathbf{z}_S) =$$

$$\sum_{s=0}^{N} \frac{1}{Z_s} \left[\sum_{\delta=0}^{\Delta} \mathrm{N}(\delta|\mu_\delta, \lambda_\delta) \left(\prod_{\substack{m=0 \\ m \neq n}}^{N_m} \sum_{r=0}^{N_r} \mathrm{N}(\mathbf{z}_{sm}|\mu_{mr}^{\delta}, \boldsymbol{\Lambda}_{mr}^{\delta}) \mathrm{Cat}(r|\theta_m) \right) \right.$$

$$\left. \times \mathrm{Cat}(j|\theta_n) \mathrm{N}(\mathbf{z}_{sn}|\mu_{nj}^{\delta}, \boldsymbol{\Lambda}_{nj}^{\delta}) \frac{1}{2} \mathrm{U}_\delta \left({\boldsymbol{\Lambda}_{nj}^{\delta}}^{-1} - (\mathbf{z}_{sn} - \mu_{nj}^{\delta})(\mathbf{z}_{sn} - \mu_{nj}^{\delta})^T \right) \right]$$

$$+ (\alpha - \frac{k}{2}) \boldsymbol{\Lambda}_{nj}^{-1} - \beta^0 - \frac{n_0}{2} (\mu_{nj} - \mu_{nj}^0)(\mu_{nj} - \mu_{nj}^0)^T \quad (4)$$

for the component covariances. The derivatives with respect to the diagonal of the matrix D_{mr} as defined in the main text are given by the diagonal of

$$\mathrm{G}^{-1} (\frac{\mathrm{d}}{\mathrm{d}\boldsymbol{\Lambda}_{nj}} \log p(M, L, \theta, \delta_1, \ldots, \delta_S | \mathbf{z}_1, \ldots, \mathbf{z}_S)) \mathrm{G}^{-1}.$$

In these derivatives,

$$Z_s = \sum_{\delta=0}^{\Delta} \mathrm{N}(\delta|\mu_\delta, \lambda_\delta) \prod_{m=0}^{N_m} \sum_{r=0}^{N_r} \mathrm{N}(\mathbf{z}_{sm}|\mu_{mr}^{\delta}, \boldsymbol{\Lambda}_{mr}^{\delta}) \mathrm{Cat}(r|\theta_m). \quad (5)$$

The notation $\mathrm{U}_\delta(\mathbf{x})$ is used to indicate that the lower-dimensional vector $\mathbf{x}$ is zero-padded to match a longer vector in the way that is appropriate for displacement δ.

3 Details of the shape model

After the intensity models for all vertices have been trained, we need to train the shape part of the model. As explained in the main text, this is done using the displacements found in the training data. These are found by simply selecting the most probable integer displacements given the MAP estimates of the parameters of the intensity models. The shape model is a simple multivariate normal distribution and we can use the standard inferential process as given by, for example, Bernardo and Smith (1994) to train it. This implies that we need to use a Normal-Wishart prior for the mean and precision parameters of the distribution. We are interested in the posterior predictive distribution

$$p(\delta|\delta_0^z, \ldots, \delta_{N_t}^z) = \int_{\mu^s} \int_{\mathbf{\Lambda}^s} p(\delta|\mu^s, \mathbf{\Lambda}^s) p(\mu^s, \mathbf{\Lambda}^s|\delta_0^z, \ldots, \delta_{N_t}^z) \mathrm{d}\mu^s \mathrm{d}\mathbf{\Lambda}^s \tag{6}$$

where $\delta_0^z, \ldots, \delta_{N_t}^z$ denote the displacements observed in the training data and where

$$p(\mu^s, \mathbf{\Lambda}^s|\delta_0^z, \ldots, \delta_{N_t}^z) = \frac{p(\delta_0^z, \ldots, \delta_{N_t}^z|\mu^s, \mathbf{\Lambda}^s) p(\mu^s, \mathbf{\Lambda}^s)}{p(\delta_0^z, \ldots, \delta_{N_t}^z)} \tag{7}$$

with the Normal-Wishart prior $p(\mu^s, \mathbf{\Lambda}^s) = \mathrm{N}_N(\mu_0^s, n_0^s \mathbf{\Lambda}^s) \mathrm{Wi}_N(\mathbf{\Lambda}^s|\alpha, \beta)$. The standard result is:

$$p(\delta|\delta_0^z, \ldots, \delta_{N_t}^z) = \mathrm{St}_N\left(\delta|\mu_t^s, \mathbf{\Lambda}_t^s, 2\alpha_t\right), \tag{8}$$

where N_t is the number of training datasets and with

$$\begin{aligned}
\mu_t^s &= \frac{n_0 \mu_0^s + N_t \overline{\delta^t}}{n_0 + N_t} \\
\mathbf{\Lambda}_t^s &= \frac{n_0 + N_t}{n_0 + N_t + 1} \alpha_t (\beta_t^s)^{-1} \\
\beta_t &= \beta + \frac{1}{2}\left(\mathrm{S} + \frac{N_t n_0}{N_t + n_0} (\mu_0 - \overline{\delta^z})(\mu_0 - \overline{\delta^z})^T\right) \\
\alpha_t &= \alpha + \frac{1}{2} N_t - \frac{1}{2}(N - 1),
\end{aligned}$$

where $\overline{\delta^z} = \frac{1}{N_t} \sum_{j=1}^{N_t} \delta_j^z$ and $\mathrm{S} = \sum_{j=1}^{N_t} (\delta_j^z - \overline{\delta^z})(\delta_j^z - \overline{\delta^z})^T$. As before, N is the number of vertices, i.e. the dimensionality of the distribution.

To produce a segmentation for a new dataset, we need to find the MAP estimate of δ. We use an approach similar to the iterated conditional modes algorithm (Besag, 1986). To find the MAP estimate for δ, we sequentially find maxima of the conditional distributions of its components δ_i given the other components. This procedure is repeated until convergence. The conditional probability of the displacement at a single vertex is the product of the conditional distribution of the shape model given all other components and the intensity model for that vertex. Up to a multiplicative constant, the conditional probability for vertex i is

$$p(\delta_i|\delta_{i^c}, Y_i, M_i, L_i, \theta_i, \mu_i^s, \mathbf{\Lambda}^s) \propto \mathrm{St}(\delta_i|\mu_{\mathrm{i}|\mathrm{i}^c}, \lambda_{\mathrm{i}|\mathrm{i}^c}, \alpha_{\mathrm{i}|\mathrm{i}^c}) p(Y_i|\delta_i, M_i, L_i, \theta_i), \tag{9}$$

where the subscript i^c denotes all components except i and the second factor on the right-hand side is the intensity model for vertex i. The parameters of this distribution are

$$\mu^s_{i|i^c} = \mu_i - (\Lambda^s_{ii})^{-1}\mathbf{\Lambda}^s_{ii^c}(\delta_{i^c} - \mu^s_{i^c}) \tag{10}$$

$$\Lambda^s_{i|i^c} = \Lambda^s_{ii}\left[\frac{\alpha + N - 1}{\alpha + (\delta_{i^c} - \mu^s_{i^c})(\mathbf{\Sigma}^s_{i^c i^c})^{-1}(\delta_{i^c} - \mu^s_{i^c})^T}\right] \tag{11}$$

$$\alpha_{i|i^c} = \alpha + N - 1, \tag{12}$$

where $\mathbf{\Sigma}^s_{i^c i^c} = ((\mathbf{\Lambda}^s)^{-1})_{i^c i^c}$. This distribution is a consequence of the conditional distributions of a subset of the multivariate Student distribution given the other part, as described in, for example, Bernardo and Smith (1994).

The intensity models are only defined for integer displacements. In practice, we evaluate both the shape and intensity model factors at the points corresponding to the integer values of δ_i and optimise discretely. There is a possibility that the final segmentation mesh self-intersects. As the reference mesh is constructed in such a way that there are no self-intersections, we remove them from the final result by incrementally deforming a mesh from the reference shape to the fitted mesh. At each small increment we use VTK's `vtkSelectEnclosedPoints` filter to test, for each vertex, whether it can be updated without generating a self-intersection. If the proposed displacement moves the vertex inwards with respect to the local surface then we know that the new point should be on the inside of the undeformed mesh (the mesh as it was before applying this update). If this proposed location is reported as being outside the mesh then it must have passed through some other part of the mesh and generated a self-intersection, in which case this point is held fixed. Otherwise, the proposed update is applied. Self-intersections for vertices that are being moved outward with respect to the reference mesh are detected in a similar fashion, but rejected if the proposed location is inside the undeformed mesh.

References

Bernardo, J, Smith, A (1994). Bayesian Theory. John Wiley & Sons.

Besag, J (1986). *On the Statistical Analysis of Dirty Pictures.* J R Stat Soc Ser B, 48(3):259–302.
